# Zinc as Allosteric Ion Channel Modulator: Ionotropic Receptors as Metalloproteins

**DOI:** 10.3390/ijms17071059

**Published:** 2016-07-02

**Authors:** Francisco Andrés Peralta, Juan Pablo Huidobro-Toro

**Affiliations:** Laboratorio de Farmacología de Nucleótidos, Laboratorio de Farmacología, Departamento de Biología, Facultad de Química y Biología, y Centro para el Desarrollo de Nanociencias y Nanotecnología (CEDENNA), Universidad de Santiago de Chile, Alameda Libertador B. O’Higgins, 3363 Santiago, Chile; francisco.andres.peralta@gmail.com

**Keywords:** zinc coordination, protein zinc ligands, zinc and ionotropic receptors, zinc allosteric modulator, zinc-activated channel, zinc coordination sphere

## Abstract

Zinc is an essential metal to life. This transition metal is a structural component of many proteins and is actively involved in the catalytic activity of cell enzymes. In either case, these zinc-containing proteins are metalloproteins. However, the amino acid residues that serve as ligands for metal coordination are not necessarily the same in structural proteins compared to enzymes. While crystals of structural proteins that bind zinc reveal a higher preference for cysteine sulfhydryls rather than histidine imidazole rings, catalytic enzymes reveal the opposite, i.e., a greater preference for the histidines over cysteines for catalysis, plus the influence of carboxylic acids. Based on this paradigm, we reviewed the putative ligands of zinc in ionotropic receptors, where zinc has been described as an allosteric modulator of channel receptors. Although these receptors do not strictly qualify as metalloproteins since they do not normally bind zinc in structural domains, they do transitorily bind zinc at allosteric sites, modifying transiently the receptor channel’s ion permeability. The present contribution summarizes current information showing that zinc allosteric modulation of receptor channels occurs by the preferential metal coordination to imidazole rings as well as to the sulfhydryl groups of cysteine in addition to the carboxyl group of acid residues, as with enzymes and catalysis. It is remarkable that most channels, either voltage-sensitive or transmitter-gated receptor channels, are susceptible to zinc modulation either as positive or negative regulators.

## 1. Introduction

Are ion channels metalloproteins? Some may qualify as such, but most ion channels activated by transmitters or agonists are not essentially metalloproteins, since they do not often contain stably bound metal(s), as occurs with enzymes or structural proteins [1]. However, receptor channels are modulated allosterically by trace metals like zinc, copper and other transition metals [2]. During the fraction of time that the channel is allosterically modulated, metals coordinate at a specific binding pocket of the receptor channel, changing its conformation and biological properties. This interaction is reversible; an indication that once the metal dissociates from the channel binding site, the native receptor conformation is recreated and susceptible to a subsequent allosteric regulation. Molecular dynamics consistently demonstrate that metal binding to the receptor channel changes the physical conformation of the protein, modifying the channel’s properties, such as its affinity for its signaling molecule, the conductance, and its single channel properties [3,4]. Therefore, in the short lapse that ion channels coordinate trace metals, as occurs in allosteric modulation, we raise the hypothesis that the channel is a true and functional metalloprotein.

This essay focuses on ion channels susceptible to trace metal regulation. We organized this essay addressing first aspects of zinc coordination chemistry and its geometry in diverse crystallized proteins, including enzymes, structural proteins, or the case of a crystallized ion channel. Next, we attempt to identify the structural determinants of the zinc coordination ligands in allosteric modulator sites of multiple receptor channels. The final goal of this area of cell biology, which amalgamates with structural biology, aims at sculpting the zinc coordination sphere involved in neuronal communication. Along the review we also address relevant issues on zinc physiology as an essential life trace metal. Although we devote special attention to the family of ion channels gated by agonists, we have not overlooked zinc interactions with the voltage-sensitive channels which are physiologically as relevant as the agonist-gated receptor channels. We need to underline that while our laboratory has systematically contributed to the pharmacology of ATP-gated channels, also known as P2X receptors, a particularly demanding effort was made to expand this review to encompass thoroughly the three classes of agonist-gated zinc-sensitive ion channels. Several recent research papers and revisions [5,6,7,8,9,10] have authoritatively addressed the relevance of Zn(II) in structural proteins as well as its role in enzyme catalysis, but to date, no review has addressed in depth the role of zinc as an ion channel regulator.

### 1.1. Zinc Background and Relevant Chemical Principles

Zinc is essential for life; it is a constituent of enzymes, involving multiple types of catalytic effects including peptidases, dehydrogenases or phosphatases, catalase, carbonic anhydrase, or dismutase activities, among other actions; therefore, it is identified as an essential human diet nutrient. This metal plays a well-recognized role in metabolic pathways, it is a component of structural proteins and transcriptional factors, and a renowned allosteric modulator; its relative importance to life is supported by a set of at least 24 zinc transporters comprising external and internal cell membrane zinc carriers.

In view of its relatively low human plasma (0.7 mg/L) or body (10–100 µg/g body weight) concentration, zinc is considered a trace metal in the body compared to sodium, calcium or potassium, whose concentrations in the human plasma or body are three to four orders of magnitude higher. Zinc body concentration is second to iron, following closely its planetary metal distribution [11]. In agreement with its accumulation in the body, zinc is the second most abundant transition and post-transition metal in the Earth’s cortex, with a concentration that ranges between 40–110 µg/g. In the peridotitic and basaltic komalities of South Africa [12] and in the surface sediments of the Calabar River in Nigeria [13], zinc was found in greater abundance than copper and cobalt, but less than iron and nickel, an indication of its ample distribution in ancestral geological formations. In this regard, considering its widespread distribution in fruits, vegetables or seeds, it is unlikely that humans, eating a normal diet, will develop a zinc deficiency.

Zinc is a transition metal belonging to the 12th group in the periodic table; it has an [Ar] 3d^10^ 4s^2^ electronic configuration with a single oxidation state Zn^2+^, so it is unlikely to participate actively in redox processes, although there is consensus that zinc does participate actively in enzymatic catalysis. Zn^2+^ is a frontier Lewis acid, allowing its coordination with “donor” atoms, and it has several coordination states ranging from two to six. It has an atomic radius of 1.4 Å, smaller than iron, accounting for its low coordination number.

### 1.2. Zinc Coordination in Proteins

**Figure 1:**
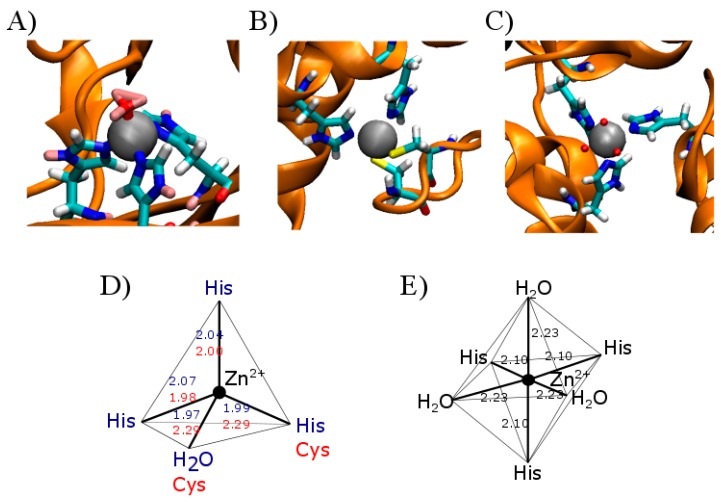
Representative examples of zinc-containing proteins and the representation of their corresponding metal coordination geometries. The illustration shows a representation of the zinc binding pockets of two classic metalloproteins. (**A**) shows the zinc binding motif in carbonic anhydrase II (PDB: 3KKX) and its corresponding coordination sphere (**D**) in blue, including the length of the coordination bonds in angstroms; (**B**) represents a zinc finger motif present in the human enhancer binding protein MBP-1 (PDB:1BBO) with its corresponding coordination sphere (**D**) in red, including binding lengths; (**C**) represents hexameric human insulin (PDB: 1MCO) with its Zn coordinating sphere (**E**) and the corresponding atom binding lengths. The light blue ball in (**A**–**C**) represents the zinc atom; dark blue represents the imidazole nitrogen involved in the coordination sphere, while red represents the oxygen, and yellow the sulfur atoms interacting with zinc. The interactions are shown by the dashed line in light blue. In (**D**,**E**) the atomic distances between zinc and its coordination sphere are indicated in angstroms. Blue numbers in (**D**) apply to (**A**), red in (**D**) apply to (**B**) and black in (**E**) correspond to (**C**) as detailed.

It has long been known that Zn(II) coordinates with proteins; it is an integral component of the catalytic site of both enzyme structural proteins as well as the archetypical “zinc fingers” [5]. As an example of conventional Zn(II) interactions with proteins, Figure 1 shows three classical examples of Zn(II) complexes with its coordination spheres and corresponding geometries. Carbonic anhydrase II is a representative example of a zinc-containing enzyme which catalyzes the conversion of CO_2_ plus H_2_O to ${\text{HCO}}_{3}^{-}$; the enzyme requires the participation of an enzyme–Zn–OH complex (Figure 1A). For this purpose, Zn(II) coordinates with three imidazole rings of key histidine (His) residues; the fourth ligand is water, allowing the formation of a tetrahedral coordination sphere [14]. Figure 1B shows a key structural motif of a Zn(II) metalloprotein which contains a relatively conserved structural domain for the metal, which corresponds to a transcription factor that binds to other proteins and ultimately to specific DNA sequences [15]. Finally, Figure 1C includes the case of hexameric insulin as an example of Zn(II) adopting an octahedral coordination, as occurs in β-pancreatic cells. In the latter case, three His residues, corresponding to three different insulin subunits, participate as a ligand in the octahedral coordination sphere. In addition to the imidazole rings, three water molecules adapt the stable octahedral geometry [16]. Although this octahedral coordination complex is not common in proteins, it must always be considered as a plausible ligand involved in the metal coordination shell.

### 1.3. Zinc Coordination in Proteins: Residues Involved in Structural versus Catalytic Sites

The dominant view until the 1970s assumed that Zn(II) coordinates with proteins, mainly—albeit not exclusively—with cysteine (Cys) sulfhydryl groups or through histidine (His) imidazole rings. Only a minor contribution was anticipated for glutamine (Gln), glycine (Gly), threonine (Thr), serine (Ser), alanine (Ala), valine (Val), leucine (Leu), tryptophan (Trp), proline (Pro), cystine (S–S), or glutamate (Glu), residues which were admitted to play a role in Zn(II) coordination only under restricted and/or distinct cases [17]. This notion has changed over the years; in fact, by the 1990s, structural studies based on crystallized Zn(II) metalloproteins indicated that the imidazole ring of His is the most prevalent zinc ligand in proteins, followed by Cys and occasionally by aspartate (Asp) and/or glutamate (Glu) [18].

**Figure 2:**
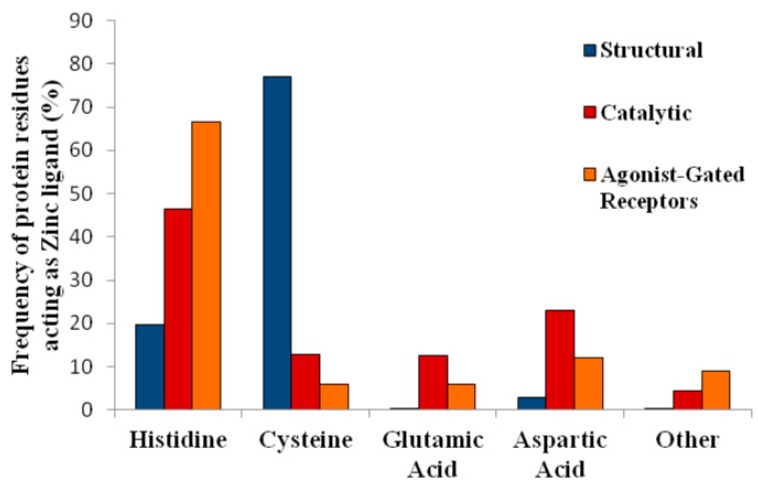
Distribution of amino acids acting as Zn(II) ligands in structural proteins, enzymes, and agonist-gated receptors. The frequency of zinc ligand amino acids was analyzed in 10,931 sites of 4882 proteins with structural or catalytic activity. The results are expressed separately for each protein regardless of its functions. The data for the structural and catalytic sites in proteins was extracted from Andreini et al. [5], while the data for 14 agonist-gated receptors is further summarized in Table 1. Currently, only one crystalline structure is available for a receptor-gated channel with its allosteric modulator zinc [20].

Interestingly, the increasing number of crystallized proteins containing Zn(II) allows dissection of the involvement of different amino acid residues in the metal coordination sphere of Zn(II) when contrasting structural vs. catalytic proteins and within the catalytic function. For example, a large proportion of Zn–O bonds were found when analyzing isomerases, in contrast to transferases and ligases, which have a higher prevalence of Zn–S bonds. Finally, lyases have a higher proportion of Zn–N bond interactions [9,19] compared to the other enzymatic functions. In full support of this proposal, there is consensus that the Zn(II) coordination sphere in proteins is primarily composed of sulfur, oxygen, and nitrogen atoms [9], and the main Zn(II) coordinating ligands are therefore Cys, His, Asp, Glu and water. Minor metal ligands include other amino acids such as asparagine (Asn), Ser, Thr, tyrosine (Tyr), Gln, methionine (Met), arginine (Arg) or Trp [8,9,19]. Altogether, and in agreement with the literature and Alberts et al. [18], Cys residues are more prevalent in the zinc coordination sphere of structural proteins, while His are more frequently found in catalytic sites [5]. Figure 2 summarizes these data comparing the distribution frequency of several amino acid residues determined to be involved as zinc ligands in structural vs. catalytic and receptor-gated channels.

In summation, the frequency of Cys, His Asp or Glu as zinc ligands in structural vs. catalytic enzymes or receptor channels is not the same. While the Zn(II) coordination sphere of structural proteins comprises primarily Cys, the metal coordination shell of enzymes relies essentially on His [5]. The reasons underpinning the differences of structural proteins vs. enzymes and the physiological implications and atomic determinants of the zinc coordination sphere in these proteins is as yet unknown, but it is exciting to understand these variations. Further investigations will allow shaping the metal coordination sphere at the purported allosteric modulator sites in channel receptors vs. enzymes or structural proteins and clearly define their roles in metal coordination.

### 1.4. Supporting Physicochemical Principles for the Zinc Coordination Sphere

The lengths of the metal coordination bonds determined using simple molecules as standard models are: Zn–N 1.7–2.4 Å, Zn–S 2.0–2.5 Å, and Zn–O 1.5–2.5 Å, with the latter value including mono- and bidentate carboxylates [8,19]. Reevaluating and deriving the zinc complex distances by two different methods, Amin et al. [35] concluded that the Zn–O complex distances were 1.721 and 1.710 Å, 2.077 and 2.067 Å for Zn–S, and 1.955 and 1.939 Å for Zn–NH_3_ [35]; the values obtained were surprisingly similar regardless of the methodology used.

When the stability constants of several amino acids–Zn(II) complexes in solution were evaluated, the most stable complex was the Cys–Zn, followed by His–Zn, and Glu–Zn [17]. This study excluded Asp complexes. A problem with these determinations is that the amino acid solution may consider for the metal complex formation the carboxyl group that is involved in the protein peptide bond (this is a group with high affinity for many metal ions). Taking advantage of this caveat, Trzaskowski et al. [36] calculated the interaction affinity between zinc and the several protein residues, in addition to reviewing the bond distance and geometry conformation for each amino acid analyzed. The results convincingly showed that the zinc affinity follows the following order of bond energies: Cys > His > Asp/Glu [36]. These data are summarized in Table 2, showing the relative energy calculated for each interaction, the bond distances, and the geometry of the zinc complexes. These results fully support the notion that Zn(II) complexes with N or S ligands are more stable than those with O ligands in the protein coordination spheres [37]. Based on these physicochemical principles, we propose that the sites involved in the more stable coordination complex should correlate with higher metal binding affinity sites likely as with metal sites in structural protein. The testing of this hypothesis in ion channels awaits experimental confirmation.

In proteins, four different geometries are associated to the zinc coordination sphere; these largely depend on the metal coordination number (4, 5 and 6). The most abundant stable conformation for a zinc coordination of four is tetrahedral, followed very closely by the square-based pyramidal or the trigonal bipyramidal geometries, both Zn(II) complexes are equally frequent for coordination five. Lastly, the octahedral geometry is exclusively for coordination number six [8,18]. Examples of the geometry and the coordination complexes of zinc-ligands are illustrated in Figure 1.

Since the electronic configuration of zinc is [Ar] 4s^2^3d^10^, the metal is diamagnetic because all its electrons are paired. Since the d shell is full, zinc has no crystal field stabilization [38], and therefore the complex stabilization is dominated by the size and the charge of the metal and ligands.

## 2. Significance of Zinc Homeostasis; Role of Metallothioneines in Human Metal Determinations

Zinc homeostasis has been extensively investigated; several excellent reviews are available [1,10,39]. Its distribution in mammals varies from 10 to 100 µg/g of wet tissue [40]. Plasma metal concentration is approximately 73 µg/100 mL, urine concentration approximately 60 µg/100 mL, while total blood concentration is approximately 570 µg/100 mL [41]. Due to the large blood reservoir of zinc, the metal bound to albumin is meaningful and in dynamic equilibrium with the tissues; the plasma albumin metal Kd is 45 nM [1].

The binding of zinc to metalloproteins occurs inside cells, where the metal articulates its real biological significance as part of multiple proteins. Since the metal does not freely diffuse into or across cells; it enters by several mechanisms among which membrane transporters are essential and play a key role in the metal homeostasis. The multiple metal carriers will be detailed to a larger extent in a next paragraph. In the brain, free extracellular zinc concentrations oscillate between 1–10 nM [42], a value considered relatively low compared to the plasma metal concentration. This difference may be accounted in part by its rapid transport inside cells mediated by selective metal carriers [7,43]. Its intracellular distribution is dynamically regulated by several cytosolic proteins and membrane transporters as muffling reactions [10]. For this purpose, metallothioneines are strategic, since these proteins bind with high affinity to heavy metals, including zinc, due to the unusually high proportion of Cys in their primary sequence. These proteins are highly regulated; within hours of an organism’s exposure to toxic metal environments, their gene expression is increased. It is currently thought that these metal sequestering proteins constitute an adaptive regulatory mechanism developed as a defense strategy and presented both by animal and plant cells. Zinc metallothioneine binding is vigorous; these proteins control free intracellular metal levels, buffering toxic concentrations both inside or outside cells [44]. In primary cultured neurons, zinc shows a diffuse distribution; the metal is found in the soma and is enriched in the nucleus, and both the soma and the processes of cortical neurons appear to have the same ability to concentrate the metal [45]. Inside neurons, zinc is stored particularly in synaptic vesicles [7]. The metal is co-released together with the neurotransmitter, particularly in glutamatergic neurons that have their soma in the cerebral cortex or in limbic structures, as in the case of Mossy fibers [42]. Therefore, it may be anticipated that zinc levels rise in the synaptic space parallel to the strength of the synapsis. In sensory neurons Aedo et al. showed for the first time that zinc modulates the spontaneous firing rate of the olfactory epithelium of the Chilean toad (*Caudiververa caudiververa*); the authors described a biphasic response characterized by an initial increment of the firing rate followed by a reduction in the firing rate of olfactory cells. The maximum firing rate was attained with 10 µM zinc; however, zinc concentrations 50 times larger consistently decreased the basal firing rate [46], evidencing not only a biphasic effect of the metal, but its influence on basal effects. This study provided strong insights that zinc influences neuronal excitability, since the metal per se did not depolarize membranes at nano or low micromolar concentrations, highlighting its role in mediating, or at least modulating, cerebral activity.

In the body, zinc is not restricted to cells or cell compartments. The metal has also been found in renal calculi in the form of apatite or oxalate salts, accounting for 1270 and 34 ppm, respectively [47]. This observation is interesting, since zinc and copper are found in comparable plasma concentrations, with slightly lower levels than iron in human serum [48] or brain [47]. However, no copper is found in either kidney or gallbladder stones.

### 2.1. Zinc Storage Mediated by Multiple Metal Transporters

**Figure 3:**
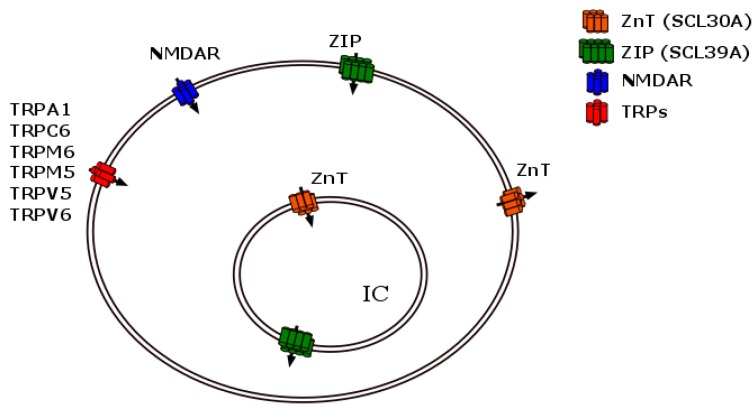
Extra and intracellular zinc transporters. The graphic summarizes multiple zinc carriers; among them several TRP channels, NMDA receptors, and the ZIP and ZnT zinc transporter families. While the influx of zinc relies on the first three families of proteins, the efflux is mediated by the ZnT transporters. TRPs and NMDAR are exclusively localized in the extracellular membrane. Both families of zinc transporters are localized in the extra- and intracellular organelle membranes, represented in this figure by a single compartment, IC. Intracellular organelle membranes may include synaptic vesicles, lysosomes, endosomes, vesicular granules (insulin-storing vesicles) and the Golgi complex.

Zinc homeostasis is the key to cell economy; it is governed by several mechanisms that regulate metal permeability, including selective transporters or membrane channels. In humans, two types of protein carriers mobilize the metal: the SLC30 (ZnT) or the ZIP SLC39 transporters [1]. While the former ZnTs are relatively zinc selective, the latter show preference for other elements such as cobalt, manganese, cadmium, or related heavy metals. Each family of ZnTs is expressed in different subcellular locations [43], and will be described in the following sections. Other metal transport mechanisms should be considered in addition to the zinc transporters [39]. Zinc enters the soma and neuron dendrites by metal-permeable channels that include *N*-methyl-d-aspartate receptors (NMDAR), α-amino-3-hydroxy-5-methyl-4-isoxazole propionic acid receptors (AMPAR) [42], and even transient receptor potential channels (TRPs) like TRPA1, TRPC6, TRPM6, TRPM7, TRPV5, and TRPV6, or even intracellularly by TRPML1 [49]. Therefore, zinc may be concentrated intracellularly either by selective metal transporter activity or by channel permeation mediated by ligand-operated channel receptors or multiple TRPs. The multiple ways of zinc transport are summarized in Figure 3 (for an expanded and specialized recent review see contribution [50] in this same volume of metalloproteins).

#### 2.1.1. The ZnT Family of Carriers

Ten separate proteins constitute this extensive family of transporters, referred to as SCL30A 1–10, or simply ZnT1–10. Each has six transmembrane segments with a His-rich loop in the cytosolic segment between transmembrane domains IV and V [43,51]. The first ZnT and consequent isoforms were first described by Palmiter et al.; ZnT1 was cloned from rat kidney and localized in the plasma membrane; functionally, it transported zinc bidirectionally, i.e., in and out of the cell [52]. Shortly thereafter, two other ZnTs were described in intracellular membranes; while ZnT2 transports the metal into endosomes/lysosomes [53], ZnT3 concentrates it in synaptic vesicles [54], a cell compartment relevant for receptor channel activity. Subsequently, the other six ZnTs were cloned; their functional properties were duly characterized and differentiated from the other carriers based both on sequence identity and kinetics [55]. ZnT5 was cloned from human tissues and found in the Golgi apparatus; its zinc Km was 0.25 µM [56]. ZnT6, was found in mice brain restricted to the Golgi and vesicles [57]; ZnT7 was also localized in the same cell compartments [58]. ZnT8 also mobilizes zinc to vesicles [59]. ZnT9 was isolated from human cells and found in cytoplasmic rather than membrane fractions [7]. ZnT10 is highly expressed in the brain, liver, small intestine, and testes, and it is limited to the Golgi apparatus [60].

#### 2.1.2. The Zinc ZIP Transporter Family

The ZIP transporters (Zrt-Irt-like proteins) were first described as iron carriers [51] and comprise a family of 14 members (SCL39A 1-14), each with eight transmembrane segments. All these proteins increase cytosolic zinc concentrations [43]. ZIP1, ZIP2, ZIP3, and ZIP4 were first cloned from *Arabidopsis thaliana*, the laboratory model of vascular plants; their apparent Km for zinc uptake is remarkably similar, with values ranging between 13.2 and 14 µM for ZIP1, 2 and 3 [61]. Taking advantage of the zinc transporters’ known sequence, Eng et al. proposed a common heavy metal binding sequence of five amino acids (HXHXH) and identified that several other previously described transporters were ZIP members [62]. Interestingly, we now know that these transporters are common to plant and animal cells, including humans. The mammalian members of the ZIP family are divided into four subfamilies: LIV1, which groups the ZIP4, 5, 6, 7, 8, 10, 12, 13, and 14 transporters; ZIPI (ZIP9); ZIPII, which includes ZIP1, ZIP2, and ZIP3 members; and ZIP11, which encompasses the gufA subfamily of transporters [7,63].

## 3. Allosteric Modulation: A Regulatory Mode in Proteins and Receptor Channels

It is more than 50 years ago that John C. and Gerhart and Arthur B. Pardee first described the case of an enzyme (aspartate transcarbamoylase, ATC) with novel regulatory properties based on feedback inhibition through the final pathway product [64]. At the same time, Monod et al. described another case of enzyme regulation by pathway metabolites; two years later, the same investigators proposed a model accounting for the observations based on the notion of allosteric sites, distant and separate from the classical enzyme substrate domain [65], localized in a different enzyme subunit. The biochemical characterization and identification of the proposed allosteric sites in different enzyme subunits was fully supported by the emerging allosterism theory. Over the years, it became clear that allosterism is a unique form of protein regulation extended to the global cell networking metabolism. By definition, allosteric regulators per se do not elicit enzyme activity, since regulators bind to a distant and distinct, non-catalytic, regulatory site termed an allosteric site. In the specific case of ATC, the end pathway product, cytidine triphosphate or uracil triphosphate, displaced the enzyme activity in a rightward manner, while adenosine trisphosphate displaced it to the left, consistent with the concept now popularized as negative/positive feedback regulation, respectively. By analogy, but several years later, it was first described that receptor channels are also susceptible to allosteric regulation by endogenous and exogenous compounds/drugs or metabolites. In the particular case of most ion channels, since they are multi subunit protein complexes (from three to five), there are also distinct sites, separate from the transmitter-binding site, sensitive to allosteric modulation. The most emblematical case of allosteric receptor channel modulators are the benzodiazepines, the best-known anti-anxiety drugs that interact at a positive/negative allosteric site within the GABA-A receptor [66]. In the present review we will go over the evidences supporting the role of zinc as an allosteric modulator of various ionotropic receptor channels, including P2X purinoceptors, receptors activated by extracellular ATP.

In keeping with Monod et al., nomenclature [65,67], allosteric modulators/regulators are either positive or negative. Positive modulators of ion channels increase the pore conductance current by several mechanisms leading finally to a leftward displacement of the agonist binding curve. On the contrary, negative modulators operate in the opposite manner, i.e., decreasing the affinity of receptor-gated currents by several plausible mechanisms. In the particular case of P2X purinoceptors, zinc is a positive modulator of both P2X2 and P2X4Rs. In contrast, copper is a positive modulator of the P2X2R, but a negative modulator of the P2X4R [68,69,70]. Several mechanisms may account for the molecular basis of the modulator role of these metals. Among other mechanisms, allosteric regulators may modify the access of the ligand to its binding site, increasing or decreasing the affinity of the receptor ligand, and favoring or interfering with the protein conformational changes that lead to pore opening, interacting at the pore itself either at the entrance or at intracellular pore sites, interacting with the membrane domain to modify the lipid environment and thereby the pore’s flexibility, altering the kinetics of ionotropic receptor desensitization, etc. All these mechanisms are valid and need to be properly dissected to decipher the exact location of the metal allosteric site and its corresponding sphere of coordination of trace metals, an issue that will be discussed in this review.

## 4. Ion Channels

Since ions are charged, their diffusion through cell membranes is restricted; ions do not cross freely between extra- and intracellular compartments regardless of very favorable differences in ionic composition. Concentration variation between compartments accounts for the electrochemical gradient proper of excitable membranes and life. Two main types of membrane channels are vastly expressed in nature in either animal or plant cells; all ion channels have a central pore through which ions cross biological membranes. Normally the pore is closed and therefore inaccessible to ion movement across the membranes. There are only two universal ways known to open a membrane’s pore or channel. The pore is either “gated” by a change in membrane voltage or the channel is “gated” by molecules that bind the channel’s orthosteric sites; such is the case of transmitter/pharmacological agonists. In short, ion channels are either voltage-gated or agonist-gated. Channels are also classified based on ionic selectivity; therefore, they may either be excitatory (if the channel is sodium or calcium-selective and favors membrane depolarization) or inhibitory if the pore is selectively permeable to chloride ions or promotes potassium efflux, leading to cell hyperpolarization. The voltage-gated channels require a change in membrane potential to open/close the channel pore; inferring that a sensory stimulus or a ligand-gated channel current must be signaled prior to the activation of these channels. In contrast, transmitter-gated channels require the release of signals such as glutamate, glycine, acetylcholine, ATP, 5-HT, or GABA. The receptor channel is specific to these messengers that selectively open cationic channels (preferentially permeable to sodium, calcium or potassium) or anionic channels (chloride) which will ultimately depolarize/hyperpolarize the cell. Table 3 and Table 4 list EC_50_ or IC_50_ values, which refer to the concentration of zinc, or an agonist, required to cause a 50% increase of the receptor-gated response or a 50% inhibition of the receptor-gated current, respectively.

### 4.1. Zinc-Induced Modulation of Voltage-Gated Channels

#### 4.1.1. K^+^ Channels

These channels constitute four main protein families widely distributed among living organisms from bacteria to humans. These channels selectively mobilize potassium from the intracellular to the extracellular space, and they exist in four main classes: (i) calcium-activated K^+^ channels; (ii) inward rectifying potassium channels (two transmembrane potassium channels); (iii) tandem pore domain potassium channels (two pore potassium channels); and (iv) voltage-gated K^+^ channels [71]. Most of these channels are inhibited by micromolar zinc; in only two cases is zinc a positive modulator within the same range of zinc concentration [72,73].

Zinc decreases the activation kinetics of the Shaker K-channel with a binding affinity of 26 µM [74] and Kv1.1, Kv1.5 (delayed rectifiers) and Kv1.4 (inactivating k-channel) channels gating, shifting the activation curves to more positive or depolarized potentials in Kv1.1 and Kv1.5, and shifting the deactivation curve to more depolarized potentials for Kv1.4 [75]. Particularly for the Kv1.5 channel, there is a reduction of the maximum conductance, reduced activation, and an accelerated deactivation [76]. This metal also modulates positively and voltage-independently other potassium channels like KCNQ5, with an EC_50_ of 21.8 µM [72]. The tandem-pore voltage-dependent K channels are two-pore channels that contribute to the cell resting membrane potential. Zinc inhibits the two-pore domain potassium TREK-1 and TASK-3 channels with IC_50_s of 659 and 12.7 µM, respectively [77], while, zinc positively modulates TREK-2 with an EC_50_ of 87.1 µM [73]. The same authors also examined the TASK-3 modulation with similar results as Gruss et al. but with a zinc IC_50_ of 25.4 µM. Moreover, zinc also activates the calcium-activated K^+^ channel Slo1 BK by switching the activation voltage to the left with an EC_50_ of 33.6 µM [78]. Except for the TREK-1 channel, all these modulations occur in the range of physiologically meaningful zinc concentrations.

#### 4.1.2. Na^+^ Channels

Although lesser in number, compared to the potassium channels, both sodium and potassium channels are vital for cell excitability. Voltage-dependent sodium channels are responsible for the depolarization of neurons and other excitable cells. Zinc modulation of Na^+^ channels was described over 30 years ago in the squid axon by Gilly and Armstrong, in experiments showing that 30 mM zinc slowed the opening of the squid axon sodium channel but did not affect its closure nor decreased its maximum conductance [79]. Twenty years later, Kuo et al. determined the zinc K_i_ value for TTX-sensitive sodium channels in dorsal root ganglion neurons. The K_i_ values ranged between 291 and 363 µM for inward currents, and between 578 and 1134 µM for the outward currents [80].

#### 4.1.3. Ca^2+^ Channels

Calcium channels are versatile and are expressed in all cells from unicellular organisms to plants and animals. These channels are divided into two major types, the high voltage activated and the low voltage activated channels, since these types of channels are not known to be directly activated by physiological ligands. One of these channel subtypes is fundamental for the release of the neurotransmitter upon nerve-ending depolarization, or the expression of the ventricular action potential in the heart or the excitability of smooth muscles [81]. Compared to potassium channels, few of these calcium channels have been thoroughly studied in the presence of zinc. This metal modulates negatively the heart T-type Ca^2+^ channels with zinc IC_50_s of 81.7, 0.78 and 158.6 µM [82] or 196.1, 24.1 and 152.2 µM [83] for Cav3.1, Cav3.2 and Cav3.3, respectively. Regarding the N-type calcium channel, mainly expressed in neurons, zinc modulates Cav2.2 with an IC_50_ of 98 µM, the P/Q-type Cav2.1 with an IC_50_ of 110 µM, the R-type Cav2.3 with an IC_50_ of 31.8 µM, and the L-type Ca^2+^ channels (expressed mainly in smooth muscle cells) Cav1.2 with an IC_50_ of 10.9 [83] or 18.4 µM; and Cav1.3 with an IC_50_ of 34.1 µM [84]. In addition, intracellular zinc blocks the calcium-activated currents in the calcium-activated chloride channel (TMEM16A) with an IC_50_ of 12.5 µM [85].

#### 4.1.4. H^+^ Channels

Voltage-gated proton channels open upon depolarization but this opening depends on the membrane’s pH gradient. They are proton selective and have low unitary conductance [86]. The zinc Kd for the H^+^ was 16 µM, determined in helix neurons when one zinc binds to this receptor channel [87]. Zinc inhibits the HV1 currents in HEK293 cells with an IC_50_ of about 2 µM [88]. The zinc coordination sphere was examined in homomeric HV1 and identified two metal binding His (H140 and H193); the association between monomers coordinates the zinc, preventing proton movement and channel opening [89].

#### 4.1.5. Cl^−^ Channels

Chloride channels are very important for the maintenance of cell volume, lysosomal acidification, cell membrane potential, and repolarization, among other functions [90]. These channels promote the entrance of Cl^−^ into the cell and therefore produce cell hyperpolarization. ClC0 was the first chloride channel cloned in this family that comprises nine members in humans [91]. As expected, Cl^−^ channels may also be modulated by the metal; the zinc EC_50_ for the negative zinc modulation of ClC0 ranges between 1−3 µM [92].

The modulator effect of zinc in a battery of voltage-dependent channels is summarized in Table 3, together with outstanding features that characterize these channels. As already indicated, the vast majority of these channels is negatively modulated by zinc; only two potassium channels (TREK-2 and KCNQ5) are positively modulated. Interestingly, one of the voltage-dependent channels was reported to be activated by Zn^2+^, a channel termed ZAC, meaning zinc-activated channel [93]. This later issue is of profound neurobiological interest since the metal in this particular case switches from a modulator to a ligand that opens the channel per se, a novel and at the same time exciting modality of channel activation.

### 4.2. Zinc-Induced Modulation of Agonist-Gated Receptors

These channels require an extracellular signal or messenger to activate the receptor that operates the channel; these molecules generally are neurotransmitters, drugs or natural products, small molecules, or protons. Signal molecules act as ionotropic receptor agonists, since they bind at the receptor’s orthosteric site eliciting a chain of protein conformational changes leading to the opening of the receptor channel pore. Ionotropic receptors, as the voltage-gated channels, are either excitatory or inhibitory, depending on the nature of the current conducted by the channel. Excitatory channels are related to the influx of cations, likely sodium or calcium channels which depolarize the membrane. In contrast, the influx of chloride anions is related to inhibitory channels that hyperpolarize the cell membrane potential. Potassium channels also cause membrane hyperpolarization agents eliciting the efflux of positive charges from the cell.

Structurally, ion channels are multiunit proteins composed of either five, four, or three subunits giving rise to the family of pentamer, tetramer, or trimer ionotropic receptors, respectively. Whereas the nicotine cholinergic, or the 5-HT3 receptors are excitatory pentamer channels (permeable essentially to sodium with a minor and variable calcium component), the GABA-A and the glycine receptors are also pentamers, but elicit inhibitory currents, due to chloride conductance. The family of glutamate receptor subtypes is composed of three tetramer members, and is the most abundantly brain expressed excitatory receptor. Finally, trimer receptors include the purinoceptors (ATP), proton-sensitive (ASIC) or amiloride-sensitive sodium channels (ENAC), all excitatory in nature. Since these channels are composed of multiple subunits, ionotropic receptors are generally heteromeric in nature, meaning that the channel is composed of several different subunits, as opposed to homomeric which are repeats of a single subunit. For example, nicotinic receptors at the neuromuscular junction are structured by α, β, γ and δ subunits. However, ganglia or the brain nicotinic receptors, which are also pentamers, are composed of only two or even one subunit, respectively. Homomeric receptors are organized of a single subunit such as the brain α7 or α9 nicotinic receptors, or the P2X7R which is composed of three single subunit repeats, depending on the nature of the receptor implied. Heterologous expression refers to the method used to assess the pharmacological properties of some channels. For example, rodent receptors are generally expressed in human cells such as the human embryonic kidney (HEK) cells, or inversely, human or rodent receptors are heterologously expressed in *Xenopus laevis* oocytes, indicating that human or rodent receptors can be easily expressed in cells from other organisms. These guidelines concerning receptor nomenclature will prove useful to follow the tables indicating the composition of the several transmitter-gated channels examined and their zinc interactions.

#### 4.2.1. GABA-A Receptors

These inhibitory receptors are inhibited by endogenous zinc in the CA3 hippocampus area [96] and, in addition, GABA-A receptor currents were inhibited by zinc in horizontal mice cells with an IC_50_ of 7.3 µM [97]. When the GABAρ1 subunit was heterologously expressed in *Xenopus* oocytes, the Kd for GABA was 1.1 µM but in the presence of 30 µM Zn^2+^ this value slightly augmented to 1.8 µM. The metal inhibited the GABA-elicited currents in GABAρ1 homomer receptors with an IC_50_ of 21.9 µM [98]; a very similar value of 20.4 µM was reported by Chang [99]. Additionally, GABA currents elicited by GABA-A receptors composed of αβγ subunits, were also antagonized by zinc, but at higher concentrations (IC_50_ of 441.3 µM in α1β2γ2 at GABA 3 µM) [99].

#### 4.2.2. Glycine Receptors

The application of zinc at low concentration potentiated the glycine-induced responses with a metal EC_50_ of 80 nM; the metal effect was biphasic since it inhibited this response with an IC_50_ of 546 µM [22] in GlyRα1 subunit heterologously expressed in HEK cells.

#### 4.2.3. Nicotinic Ach Receptors

Zinc exerts a biphasic effect on α and β heteromers; low zinc concentrations caused a positive modulation of the acetylcholine response. However, higher metal concentrations reduced the response compared to the controls with acetylcholine alone. The zinc EC_50_ was 13 µM for α2β2, 45 µM for α2β4, 47 µM for α3β4, 16 µM for α4β2, and 22 µM for α4β4; however, zinc concentrations five- to ten-fold higher elicited IC_50_ of 52 µM for α2β2, 590 µM for α2β4, 97 µM for α3β2, 3200 µM for α3β4, 440 µM for α4β2 and 510 µM for α4β4, respectively [100]. In α7 homomers, the application of zinc caused a negative modulation effect with a metal IC_50_ of 27 µM [101].

#### 4.2.4. 5-HT3 Receptors

Out of the more than 20 serotonin receptors (5-HTR) cloned, only one, the 5-HT3 subtype, is an excitatory ionotropic receptor [102]. This receptor belongs to the pentamer family of ionotropic receptors and is positively modulated by zinc. In particular, the homomeric 5-HT_3A_ receptor is positively modulated by zinc in micromolar concentrations [103].

#### 4.2.5. NMDA and Related Glutamate Receptors

These receptors are known to be negatively modulated by zinc; the regulation depends markedly on subunit assembly. In cases of heterologous co-expression of NR1 and NR2B receptors in HEK293 cells, zinc has an IC_50_ of 9.5 µM. However, when NR1 and NR2A subunits are co-expressed in HEK293 cells, zinc also causes a negative modulation mediated apparently by two sites with IC_50_s of 5 nM and 79 µM, respectively [104]. Therefore, the NR2A subunit increased 1000-fold the negative zinc modulation. This striking difference in affinity suggests that the allosteric interactions, although with the same “working” group of amino acids, should be based on a different coordination shell than those we find in traditional structural and enzymatic metalloproteins. Kainite receptors are also inhibited by zinc; homomeric or heteromeric channels containing GluR6, KA1 and KA2 subunits are inhibited by zinc with IC_50_s of 67 µM for GluR6, 1.5 and 2.1 µM for GluR6/KA1, and GluR6/KA2, respectively [105]. Zinc exhibited a biphasic effect on the homomeric GluK3 receptor, where low zinc concentrations elicited a potentiation, while higher metal concentrations caused inhibition with an EC_50_ and IC_50_ of 46 and 100 µM, respectively [20].

#### 4.2.6. H^+^ and ENAC, Na^+^ Receptors, Belonging to the Trimer Receptor Family

##### Protons Activate the Acid-Sensitive Cationic Channels (ASICs), Widespread in the Body

ASICs are modulated by zinc in a mode depending on channel subunit composition. While zinc is a positive modulator of the ASIC2a pH response, with an EC_50_ of 120 µM [23], low nM zinc inhibited the ASIC1a channel with an EC_50_ of 14.2 nM [32]. It follows that as with the NMDA and related receptors, the zinc effect is highly dependent on subunit composition. Zinc acts on an inhibitor modulator site, with metal affinities in the range of 5−10 nM.

##### The ENaC Receptor

The ENaC receptor is a fundamental sodium channel highly expressed in the kidney nephron collecting tubule which is characterized by amiloride inhibition, a prototype collecting tubule diuretic. αβγ heteromers are zinc-sensitive, with increased currents and abolishment of the sodium auto-inhibition with 100 mM extracellular sodium. The positive modulator effect of the metal was observed with EC_50_s of 1.7 [106] or 2.1 µM [24] and voltage-independence [106]. However, with 1000-fold more zinc, the effect was inverted, exhibiting a negative modulation [107] with IC_50_ of 2.1 mM zinc [24].

##### P2X Purinoceptors

Over the past 20 years, the physiology and extracellular role of P2X has been vastly recognized in almost every tissue. It is not an exaggeration to claim that ATP is a novel, although ancient, extracellular cell messenger of both animal and plant cells. The more we understand about the physiology of extracellular ATP, the more convinced we are of its paramount role in cell physiology. The P2X purinoceptors are mainly activated by extracellular ATP and comprise a family of seven receptor clones (P2X1 through P2X7). These receptors are modulated by several trace metals and particularly by zinc [2]; the modulator effect differs in the multiple P2X receptor subtypes and the modulation depends on the metal preincubation exposure. P2X1 is negatively modulated with an IC_50_ of 9.3 µM when ATP and Zn^2+^ are coapplied, but it changes to approximately 1 µM when Zn^2+^ is preapplied [108], while P2X2 is positively modulated by Zn^2+^ when Zn^2+^ and ATP are coapplied with an EC_50_ of 9.3 µM [109,110], but when Zn^2+^ is preincubated the response to ATP was potentiated below 30 µM Zn^2+^, and at higher Zn concentrations there was an inhibitory effect [110], the potentiation of ATP-activated currents were voltage independent [111]. P2X3 is potentiated with an EC50 of 10.9 µM, but when Zn^2+^ is preincubated, the potentiation turns into a bell-shaped C/R curve reaching the maximum I_ATP_ at approximately 20 µM Zn^2+^ [108]. P2X4 are also potentiated by Zn^2+^ in a voltage independent manner (EC50 of 2.4 µM) [111], but at concentrations higher than 30 µM this potentiation changed to an inhibitory form, and this effect was observed in the coapplication or preapplication of Zn to ATP [68,112,113]. P2X5 is modulated by extracellular zinc in a biphasic mode. Low metal concentrations potentiate the response to ATP, while at higher concentrations the response decreases [114]. As in the case of P2X1, P2X7 is inhibited by zinc with an IC_50_ of 11.2 [115] or 4.6 µM [33] when the receptor is expressed in HEK cells and uses di-benzoyl ATP as an agonist, and 78 µM when expressed in *Xenopus* oocytes; the receptor was challenged with ATP as an agonist and preincubated for 10 s with zinc [34].

Several P2X receptor subtypes are modulated by zinc both positively and negatively; in some cases the zinc response is biphasic. At low metal concentrations the modulatory effect is positive, an effect that shifts to a negative modulation at higher metal concentrations (see Table 4).

##### Transient Receptor Potential Channels (TRPs)

This is a very large family of channels composed of 28 mammalian members described as polymodal cell sensors. Six TRP subfamilies have been described: canonical (TRPC), vanilloid (TRPV), melastatin (TRPM), polycystin (TRPP), mucolipin (TRPML), and ankyrin (TRPA) [116]. These are not strictly receptor channels, since their activity is triggered by a variety of sensory stimuli that require our attention. Out of this large family of channels, two particular members, TRPM2 and TRPM5, are zinc-modulated. When TRPM2 was stimulated with ADP-ribose (ADPR), zinc application inhibited the channel due to inactivation of the receptor above 30 µM of extracellular zinc [117]. The application of zinc at lower concentrations inhibits the intracellular Ca^2+^-activated TRPM5 with an IC_50_ of 4.3 µM [118].

##### The Special Case of Intercellular or Hemichannels

Hemi-gap-junction channels are modulated by divalent cations, with zinc being the most potent inhibitor with an IC_50_ of 37 µM in horizontal retinal cells; the effect is calcium independent [119].

## 5. Deciphering the Zinc Binding Sites in Receptor-Gated Channels

### 5.1. Identification of Strategic Amino Acid Residues of the Metal Coordination Sphere

Although at present there are not as yet firm grounds to establish the zinc coordination sphere of most ionotropic channel-gated receptors, there has been steady progress using electrophysiology of natural (wild-type) or artificially mutated receptors expressed in several systems as a test of receptor activity. For example, zinc under 10 µM is a negative modulator of NMDA receptors in a voltage-independent manner; however, above 30 µM zinc, the receptor behaves as a voltage-dependent modulator with characteristics similar to the Mg^2+^ receptor blockade. While the modulation is not very significant for the NR1/NR2B glutamate receptor, since 80% of the receptors are inhibited with low zinc concentrations, it becomes relevant for NR1/NR2A receptors, where only 40% of the receptors were inhibited by low zinc [104]. The high affinity binding site for zinc in the NR1/NR2A receptor has been studied by point mutations and the residues most probably involved in this coordination were found to be H44, H128, K233 and E266, with a less important participation of H42 [29,30]. Chang et al. reported a zinc modulation of GABAρ1 subunits, pointing out that the metal appears to compete with GABA at 100 µM zinc; at higher concentrations, the metal appeared to be a non-competitive antagonist [99]. Wang et al. discovered that H156 is a key for the zinc modulatory effects [31]. In nicotinic receptors, a zinc coordination sphere was proposed, and there are two sites for zinc. The positive zinc modulator site involves at least four critical His residues [100]. In the case of α4β4 nicotinic receptors, it is advanced that the zinc binding site involves four amino acid residues, three of which are located in the α4 subunit and one in the β4 subunit. These strategic amino acid residues identified for zinc coordination are E59, H61 and H162 of the α subunit and H469 of the β subunit [21].

As indicated in a previous section, homomeric ASICs respond in a biphasic mode to zinc, suggesting two potential zinc binding sites. While the positive zinc modulation in the ASIC2a channel is dependent on H162 and H339 [23], the negative modulator role of the metal in the ASIC1a is dependent on K133 [32]. Additionally, and consistent with the aforementioned findings, heteromeric ASIC1a+2a receptors also respond to high metal concentrations [23], indicating perhaps that heteromeric ASICs may have a dual zinc response pattern depending on two coordinating spheres. Hypothetically, one could be located in ASIC1a and the other in ASIC2a, a thesis that should be clarified in the coming years.

Regarding TRP channels and the transport of zinc, the metal was shown to cross the cell membrane by TRP melastatin 2 channel activity [121]; the activation of TRP melastatin 7 [122] or by the basal activity of the TRP melastatin 1 channel was shown to depend on D915, an amino acid which acts as a selective divalent cation filter [49]. Therefore, it was of interest to determine the site of zinc coordination in these channels. Towards this goal, the TRPA1 channel was shown to be localized in the channel intracellular segment, and to coordinate zinc the channel requires interaction with amino acids C641, H983 and C1021 [120]. Future studies should establish the sites of metal coordination in melastatin 1, 2 and 7 channels. Interestingly, the modulation of trimeric channels such as ENaC or ASIC is different. Low zinc concentrations exhibit a positive modulator effect while higher metal concentrations elicit a negative modulation. The structural basis of this dichotomy has shown that H88 in the γ subunit abolished the inhibitory effect of zinc, while H193, H200 and H202 are involved in the positive allosteric modulator role of the metal activity in adjacent receptor subdomains [24].

It becomes apparent that in addition to the traditional Cys and His zinc ligands, and in keeping with the more recent trends listed in Figure 2, acidic amino acids such as Glu or Asp, or even basic amino acids, form part of the zinc coordination shell in agonist-gated receptors. This analysis is internally consistent with our meta-analysis and helps to expand the participation of noncanonical amino acid residues as zinc ligands in agonist-gated channels.

GlyRα1 homomeric receptors are modulated by zinc in a biphasic form and the addition of DEPC abolished both the activation and the inhibition (without affecting the glycine-induced currents), indicating that histidine residues are necessary for this modulations. Site-directed mutagenesis showed that H107 and H109 are important for Zn^2+^ inhibition, and H107 is also important for Zn^2+^ potentiation [22].

**Figure 4:**
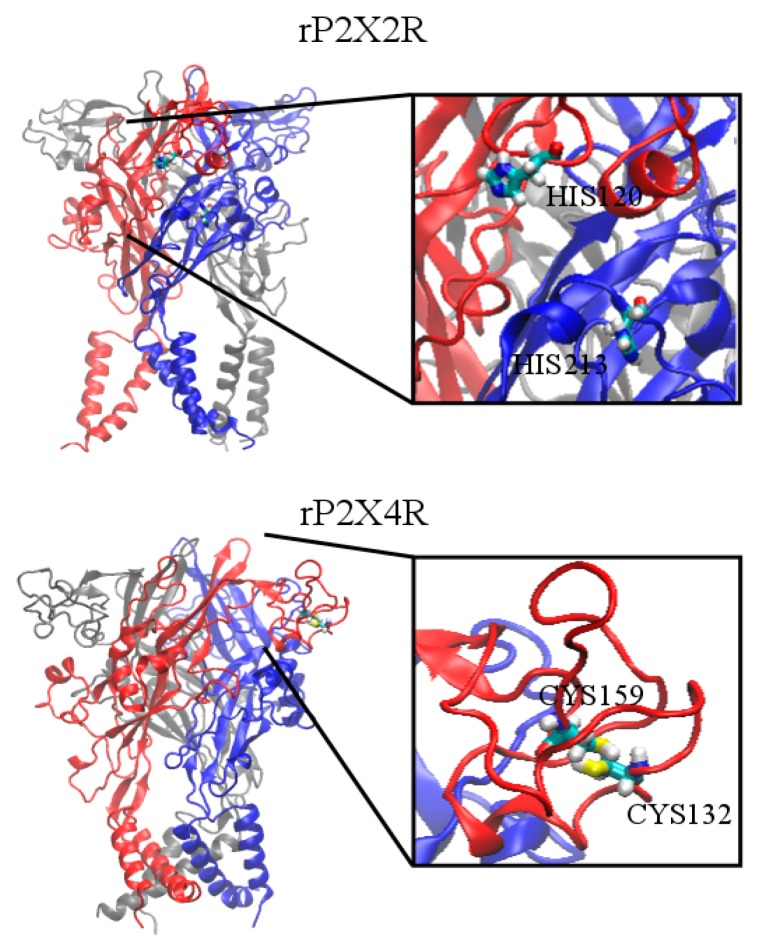
Modeling structural differences in zinc coordination between P2X receptors. The figure shows two molecular models of P2X receptors with their known zinc modulation participating residues. The upper panel represents the rat P2X2 receptor, while the lower panel represents the P2X4 receptor. In both receptor models, the three receptor subunits are represented in red, blue and gray colors. The intracellular domains were removed in the homology model based on the P2X4 receptor crystals from *Danio rerio* (zebra fish). Note the difference in the zinc coordination sphere between the two P2X receptors. While the zinc coordination in the P2X2 receptor is mediated by two key His, each localized in a different receptor subunit, in the P2X4 receptor, two Cys were identified as part of the zinc coordination sphere, both localized in the same receptor subunit.

P2X receptors are particularly attractive because of the differential modulation by zinc metal, providing the opportunity to learn from structural biology about the localization of the metal coordination sphere. While zinc is a positive zinc modulator of both P2X2 and P2X4 receptors, striking differences were found between these receptor subtypes. Two His were shown to be ligands for the zinc coordination on the P2X2 receptor (H120 and H213); moreover, a metal binding pocket was located in the interface between subunits [25,26]. See structural model presented in Figure 4. Interestingly, in the positive P2X4 receptor metal modulator role, no His was shown to constitute the zinc coordination sphere; instead, two Cys (C132 [27] and C159 [28]) were recognized as zinc ligands. Figure 4 also shows that the zinc coordination shell in the P2X4 receptor is localized intrasubunit, establishing two notable differences among receptor subtypes which share ca. 50% receptor identity. In addition, other residues in the vicinity of both Cys, such as T133 [27] are also likely to be involved in the zinc modulation. In view that zinc has a biphasic modulator effect on the P2X4 receptor, it was established that D136 and H140 are key residues required for the negative zinc modulation which occurs at higher metal concentrations [27]. Regarding the coordination of zinc at the P2X7 receptor, imidazole rings of H219, H267 [34], and H62 plus D197 [33] were identified as ligands for the negative zinc modulation.

### 5.2. Structural Evidence in Agonist-Gated Ionotropic Receptors

Crystallographic structures addressing zinc coordination in receptors or channels are critical for this purpose. For example, zinc plays a structural role in the rod cyclic nucleotide-gated channels [123] or the Shaw potassium channel Akv3.1 [124]. The only example of a crystallized ionotropic receptor channel containing zinc was detailed by Veran et al. [20] and refers to the GluK3 subunit of the kainate receptor. This study involved site-directed mutagenesis and electrophysiological assays; crystallographic results consistently conclude that the zinc coordination sphere in the homomeric receptor involves Q756, D759 and H762 in one subunit and D730 in the adjacent subunit; one or two water molecules are involved in the zinc coordination shell as well [20]. Moreover, the heteromeric GluK2/GluK3 receptor is also positively modulated by zinc; the zinc coordination shell includes D729 (in GluK2) and Q756, D759, and H762 (in GluK3) of heteromeric receptors. Therefore, the tetrameric receptor must be molded by two heterodimers of GluK2/GluK3 and the zinc binding site will be in the interphase of both subunits [20].

It becomes most exciting and gratifying to foresee that, as summarized in Figure 2 and detailed in Section 1.3, nonclassical zinc ligands are part of the metal coordination shell which encompasses acidic amino acids and even glutamine.

### 5.3. Is There a Difference in the Zinc Coordination Sphere When Comparing Positive and Negative Allosteric Modulators?

**Figure 5:**
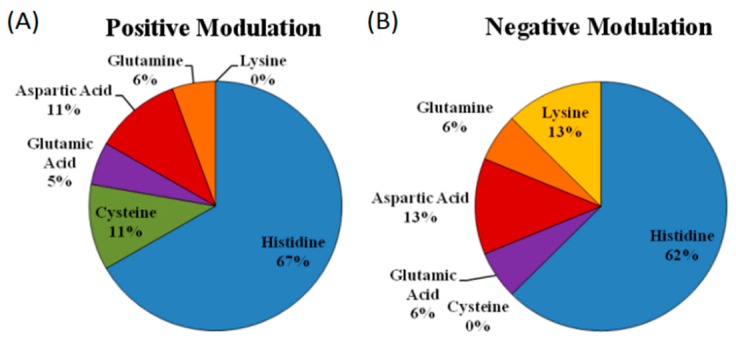
Comparison of zinc ligands in positively modulated vs. negatively modulated agonist-gated receptors. We examined 14 known receptor channels depicted in Table 1 and ascertained in these channels whether the same amino acids are part of the zinc coordination sphere in the case of positive (**A**) and negative (**B**) modulation zinc-responses. The results show that while His is the most prevalent zinc ligand in either type of allosteric modulation examined, Cys is only present in the positive modulation. No cysteines were found in the negatively zinc-modulated receptor channels. Interestingly, Lys is only a zinc ligand of the negatively zinc-modulated receptor channel, an amino acid that forms part of the zinc coordination shell which is not frequently encountered as a recognized zinc ligand.

To address this question we relied on the few examples of zinc coordination spheres determined in receptors where zinc acts as a positive or a negative allosteric regulator. The analysis of the available data revealed that the frequency of amino acids participating on the coordination sphere of zinc in allosteric modulator sites resembles more closely those involved in catalytic sites than those found in structural zinc coordination shells (Figure 2). Moreover, when we examined separately the amino residues involved in positive vs. negative zinc modulators, we consistently found that while the frequency of His residues is about the same in positive vs. negative allosteric regulators, Cys were only found in positive coordination spheres, while Lys were only found in the negative modulators (Figure 5 and Table 1). These results are surprising and quite exciting, since a priori, an equal distribution could be expected in either case. However, the data allow differentiating between residues constituting either metal coordination spheres. While Cys only appears in shells of positive modulation, lysines (Lys) are present only in negative modulation sites. Remarkably, ligands such as Asp or Gln or Glu have almost identical proportions on either mode of regulation. This analysis was made taking in account the proposed ligands for the modulation of ligand-gated ion channels presented in Table 4, but as reviewed in Section 4, zinc coordination in HV1 channels is composed of two His ligands [89], supporting the view that the zinc ligands in allosteric modulation are similar to those found in catalytic sites of proteins. To our knowledge, this is the first report distinguishing zinc ligands based on the mode of receptor regulation by allosteric modulators. It is seen that at present few cases are available for a more detailed examination of this hypothesis, but this is a trending topic that will profit from further research.

## 6. Zinc as a Ligand of Ionotropic Receptor Channels

**Figure 6:**
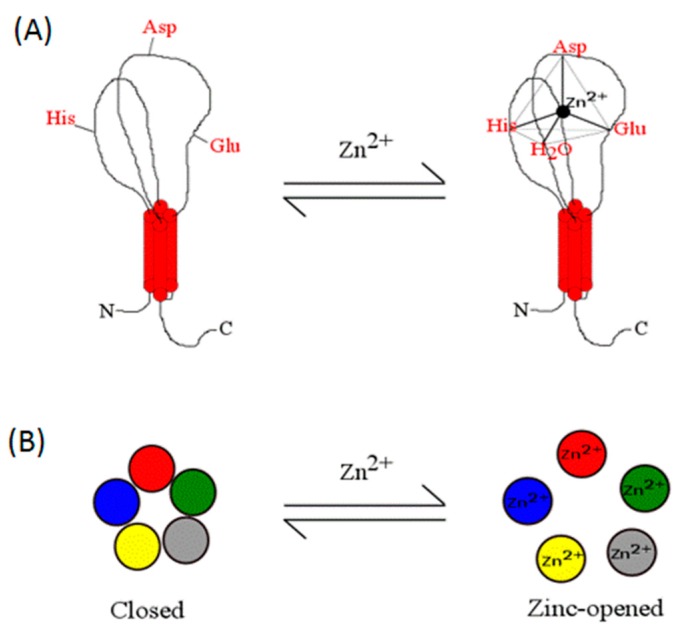
Hypothetical model of a zinc-gated receptor channel, ZAC, and its putative coordination sphere. ZAC is a member of the pentamer superfamily of ionic receptor channels; its subunit composition is likely homomeric, composed of five identical subunits with four transmembrane domains each. (**A**) Graphic of a single subunit showing putative residues involved in the zinc coordination shell. In the presence of the metal, the putative receptor coordination site binds zinc probably with tetrahedral geometry; (**B**) The circle represents the five receptor subunits which constitute the active channel; in the presence of the metal, the channel opens and elicits an excitatory inward current. Based on the proposed model, since the metal coordination sphere is intrasubunit, five zinc atoms are required to open the channel.

The exciting report by Davies et al. opened the debate as to whether zinc is an extracellular messenger that triggers selectively the opening of a cationic channel [93]; in support of this proposal, a zinc-activated channel (ZAC) was cloned. ZAC is a novel type of ionic channel belonging to the pentamer super family of Cys-loop receptors gated in a concentration-dependent manner by zinc. Its metal EC_50_ is 540 µM [93]; it is voltage-independent and exhibits spontaneous activity. Among the pharmacological features of the channel, it elicits an outward rectifier current inhibited by tubocurarine, a nicotinic receptor antagonist. This rare and curious receptor channel has several acidic amino acids and His on its extracellular domain; therefore it is not surprising to us that zinc may coordinate with some of these amino acids to form a zinc coordination complex that gates the channel. In view of our current understanding of zinc coordination with proteins, we advanced a putative model to propose how zinc coordinates with strategic metal ligands following a tetrahedral geometry. Since ZAC fits into the pentamer family of receptor channels, we anticipate that five zinc coordination shells are required to operate ZAC. A hypothetical model on how we envision the channel gating is presented in Figure 6.

Furthermore, zinc is also known to activate TRPA1 receptors by binding to the intracellular segment of the channel with a 2.3 µM EC_50_ when heterologously expressed in HEK293 cells [120]. Furthermore, zinc activated intracellularly the K_ATP_ channel Kir6.2 when expressed with SUR1 (Kd of 1.8 µM) and SUR2A (Kd of 60 µM), with the SUR subunits being responsible for this activation [95]. However, not only intracellular zinc has an effect over Kir6.2, it is also effective when applied extracellularly, where it activates the SUR1/Kir6.2 channels but slightly inhibits the SUR2A/Kir6.2 channels [95]. This extracellular activation was already observed for Kir6.2 but in RINm5f cells with a metal EC_50_ of 1.7 µM, modifying the channel open state probability [94].

This evidence is exciting, since it shows unambiguously that zinc is not only a physiologically relevant allosteric modulator, but it also plays a role as an activator or an “agonist” of classical ionotropic receptors like ZAC or the TRPA1 receptor channel, as well as voltage-gated channels.

## 7. Zinc-Associated Pathologies: A Link between Channel Receptors and Zinc Modulation

Aged patients with a metabolic syndrome have higher serum zinc concentrations than healthy volunteers [125], raising the hypothesis that zinc might be associated with pathophysiologic processes leading to major neurodegenerative diseases including Alzheimer’s and amyotrophic lateral sclerosis [42]. Particularly, in Alzheimer disease, high zinc concentrations were reported in serum and amyloid plaques; furthermore, aggregation of Aβ peptide is favored in the presence of zinc. The zinc present in Alzheimer’s plaques may derive from synaptic release from glutamatergic nerve terminals [126].

In addition, we now discuss recent data on three brain pathologies which involve zinc transporters or P2X receptors in the context that brain transmission, susceptible to zinc modulation, may be associated with signs and symptoms proper of these diseases. First, an interesting association linking schizophrenia with a mutation of ZIP8 was reported, which leads to reduced zinc accumulation in neurons, this condition causing elevated synaptic zinc concentration that will modulate negatively the NMDA receptors containing the NR2A subunit [43]. Second, Morris et al. suggest that there is an association between epilepsy and zinc channel modulation. Two lines of evidence support this proposal: first, zinc sulfate injected into rabbits produced seizures; similarly, zinc chloride increased the kainite-induced hippocampus neurotoxicity. However, when a zinc chelator was applied before the convulsing stimuli, seizure duration and electrical discharges decreased. The second line of evidence derives from protocols which showed an increased probability of kainite-induced seizures in ZnT3 KO mice and of mice raised on a low zinc diet. These mice also have larger levels of glutamate and less GABA in hippocampal extracellular fluid after kainite treatment. The zinc deficiency increased seizure susceptibility in a mice model of epilepsy, and this condition was reverted with zinc dietary supplements [127].

Finally, a decrease of blood serum zinc was recently reported in humans diagnosed with autism spectrum disorders [128,129]; interestingly, P2X4R KO mice also evidenced signs and symptoms characteristic of this brain pathology [130]. Although at present we do not fully understand the implications of these findings, the hypothesis emerges that autists either experience a reduction in brain P2X4R expression or the facilitator action of zinc on P2X4R-gated currents is not potentiated enough due to reduced zinc availability and P2X4Rs expression deficit. The opportunities of prescribing zinc as a health supplement might be worth considering as a simple way to transiently modify brain neurotransmission, improving cognitive functions.

## 8. Biological Implications and Future Perspectives

Allosterism, which originally was projected as a novel opportunity of enzymatic regulation with the obvious potential of becoming a global cell metabolic component [67], can, in our view, be successfully expanded to include almost all multi subunit proteins, including, of course, ion channels and therefore neuronal communication. This review highlights that almost all ion channels, either agonist-gated receptors or voltage-dependent, are regulated allosterically in both a positive or negative mode by zinc. Based on this premise, we anticipate that allosterism is a universal regulatory mechanism with vast implications in health and disease. Therefore, it is not surprising that the pharmaceutical industry is devoted to developing new targets for drug action based essentially on allosteric sites [131]. There is a well-documented dossier of novel chemicals for the nicotinic, NMDA, and purinergic receptors, formulated to act at allosteric sites with a strong clinical potential.

An issue that cannot be ignored refers to the relative zinc affinity when allosteric modulator sites and structural protein sites are compared; a topic that may be tackled as the “affinity problem.” We know that zinc coordination yields sites of high affinity, yet nearly all the allosteric zinc interactions described to date range within micromolar affinity, a value that might not be physiological considering that, for example, the brain extracellular free zinc concentration is in the range of 1−10 nM [42]. Estimates of extracellular zinc concentrations in the synapses raises to 30 µM concentrations [42], considering synaptic zinc release as in the case of glutamatergic/zincergic synapses. These values might be increased particularly following high frequency bursts or tetanic stimulation, as in epilepsy episodes. A proof of concept requires the analytical measurement of the synaptic metal concentrations, a challenge that awaits more precise brain cannulae coupled to new technological developments for in situ metal determinations.

It is of interest to recognize that allosteric, like orthosteric sites, recognize chemicals that were evolutionarily selected as endogenous compounds related to cell metabolism. Due to the structural homologies of their spatial configuration, these sites can be mimicked by exogenous compounds like drugs. Despite the complexity of these molecules, allosteric sites can also be activated by simple chemicals like zinc or other transition metals essential for cell metabolism.

An exciting opportunity linked to the role of allosteric modulation regards the potential therapeutic use of zinc to increase cognitive functions and related medical opportunities. In view of its essential role for life and for the nervous system, we recently demonstrated that zinc increased long-term potentiation [132], an interesting finding related to cognition and learning. Although in that paper we were not able to determine which of the many ion channels regulated by zinc were involved, several channels were raised as candidates. The pharmaceutical formulation of the metal plus vitamin supplements, as currently used, is a correct approach that may result in brain function improvement [133]. As to the use of nanostructured particles for zinc delivery to improve the pharmacokinetics of its administration, particularly its brain accessibility, it remains as an open question that deserves further experimentation. An attempt to deliver zinc as nanoparticles is an emerging issue that should receive unambiguous medical interest and attention.

## 9. Concluding Remarks

Our contention that ion channels, particularly agonist-gated receptor channels, may be considered metalloproteins is, in our mind, abundantly supported by the reviewed data, which will certainly be enriched by further experimental support. As with structural proteins and/or enzyme catalysis, zinc binds with agonist-gated channel receptors at defined sites, exhibiting classical geometries, and bonding through preferred amino acid ligands at the metal coordination sphere. Our argument that the amino acid residues involved in the metal coordination at allosteric modulation sites bear more similarities with those involved in catalysis than in structural proteins is compatible with the receptor role as transient metalloproteins that bind the metal depending on its bioavailability in the synaptic space. Furthermore, we have added that it is very likely that the coordination ligands required for positive versus negative zinc coordination may be different.

It must be concluded that almost all ion receptor channels are modulated by zinc, an indication that probably trace metal modulation is a primitive, ancient, and versatile mechanism to modulate the biophysical properties of agonist-gated channels. Insofar as little is known about the evolutionary history of chemical transmission, ATP-gated receptors are probably one of the most primordial signaling receptors known. It should be noted that all seven subtypes of ATP-gated receptors are modulated by zinc, an observation valid for almost all the P2X receptors expressed along the evolutionary species history of these receptors. Perhaps this finding is an indication that extracellular zinc was common in the extracellular life milieu and became an evolutionary advantage available to almost all receptor channels along the history of life.

## Figures and Tables

**Table 1:** Zinc ligands in ionotropic receptors.

Type of Zinc Modulation	Receptor	aa1	aa2	aa3	aa4
**Positive Modulator**	GluK3	Q756 [20]	D759 [20]	H762 [20]	D730 [20]
	NAChR α4β4	α E59 [21]	α H61 [21]	α H162 [21]	β H469 [21]
	GlyRα1	H107 [22]			
	ASIC2a	H162 [23]	H339 [23]		
	ENaC γ	H193 [24]	H200 [24]	H202 [24]	
	P2X2	H120 [25,26]	H213 [25,26]		
	P2X4	C132 [27,28]	C159 [28]		
**Negative Modulator**	NR1/NR2A	H44 [29,30]	H128 [29,30]	K233 [29]	E266 [29]
	GABAρ1	H156 [31]			
	GlyRα1	H107 [22]	H109 [22]		
	ASIC1a	K133 [32]			
	ENaC γ	H88 [24]			
	P2X4	D136 [27]	H140 [27]		
	P2X7	H62 [33]	D197 [33]	H219 [34]	H267 [34]

Please note that aa refers to amino acids identified participating in the metal coordination sphere.

**Table 2:** Characteristics of zinc interactions with single amino acid side chains.

Amino Acid	Interaction	Energy (kJ/mol) *	Distance (Å)	Geometry of the Complex ^†^
Cys	Zn–S (Cysteine)	−60.35	2.27	Tetrahedral
His	Zn–N (Imidazol)	−34.26	2.07	Octahedral
Asp, Glu	Zn–O (Carboxyl)	−4.49	2.18	Octahedral

* Theoretical values relative to water; ^†^ zinc complex geometry conformation with the corresponding amino acid and water molecules. Adapted from the reference by Bartosz Trzaskowski et al. [36].

**Table 3:** Voltage-gated ion channels modulated by zinc.

Channel Name	Subtype or Subunit	[Zn^2+^] (EC_50_ or IC_50_)
**K^+^ Channels**	Kv1.1 (NM)	N.D. [75]
	Kv1.4 (NM)	N.D. [75]
	Kv1.5 (NM)	N.D. [75]
	TREK-1 (NM)	659 µM [77]
	TREK-2 (PM)	87.1 µM [73]
	TASK-3 (NM)	12.7 µM [77]; 25.4 µM [73]
	Shaker (H4) K-channel (NM)	N.D. [74]
	Slo1 K (BK) channels (A)	33.6 µM [78]
	KCNQ5 (PM)	21.8 µM [72]
**ATP-sensitive K^+^ Channels**	Kir6.2 (A)	1.7 µM [94]
	SUR1/Kir6.2 (A)	Extracellular N.D.; Intracellular 1.8 µM [95]
	SUR2A/Kir6.2 (NM, extracellular; A, intracellular)	Extracellular N.D.; Intracellular 60 µM [95]
**Na^+^ Channels**	Nav (NM)	N.D. [79]
	TTX-sensitive Na channels (NM)	N.D. [80]
**Ca^2+^ Channels**	TMEM16A (NM)	12.5 µM [85]
	Cav1.2 (NM)	10.9 µM [83]; 18.4 µM [84]
	Cav1.3 (NM)	34.1 µM [84]
	Cav2.1 (NM)	110 µM [83]
	Cav2.2 (NM)	98.0 µM [83]
	Cav2.3 (NM)	31.8 µM [83]
	Cav3.1 (NM)	81.7 µM [82]; 196.1 µM [83]
	Cav3.2 (NM)	0.78 µM [82]; 24.1 µM [83]
	Cav3.3 (NM)	158.6 µM [82]; 15.2 µM [83]
**H^+^ Channels**	HV1 (NM)	2 µM [88]; 16 µM [87]
**Cl^−^ Channels**	ClC-0 (NM)	1–3 µM [92]
**ZAC**	ZAC (A)	540 µM [93]

NM, negative modulator; PM, positive modulator; A, activator; EC_50_ refers to the metal’s median concentration causing a 50% increase of the activated current; IC_50_ indicates the metal’s median concentration causing a 50% inhibition of the receptor-gated current; N.D., values not determined

**Table 4:** Ligand-gated ionotropic receptors modulated by zinc.

Receptor Type	Subtype or Subunit	Ligand	[Zn^2+^] (EC_50_ or IC_50_)
GABAergic	GABAρ1 (NM)	GABA 1 µM [98], GABA 3 µM [99]	21.9 µM (1 min PI) [98], 20.4 µM [99]
	GABAα1β2γ2 (NM)	GABA 3 µM [99]	441.3 µM [99]
	GABAA (NM)	GABA (WC) [96], GABA 50 µM [97]	N.D. [96], 7.3 µM [97]
Glycinergic	GlyRα1 (PM/NM)	Glycine 50 µM [22]	80 nM (PM); 546 µM (NM) [22]
Cholinergic	α7 (NM)	Acetylcholine 3 µM [101]	27 µM [101]
	α2β2 (PM/NM)	Acetylcholine 3 µM [100]	13 µM (PM); 52 µM (NM) [100]
	α2β4 (PM/NM)	Acetylcholine 3 µM [100]	45 µM (PM); 590 µM (NM) [100]
	α3β2 (NM)	Acetylcholine 3 µM [100]	97 µM [100]
	α3β4 (PM/NM)	Acetylcholine 3 µM [100]	47 µM (PM); 3200 µM (NM) [100]
	α4β2 (PM/NM)	Acetylcholine 3 µM [100]	16 µM (PM); 440 µM (NM) [100]
	α4β4 (PM/NM)	Acetylcholine 3 µM [100]	22 µM (PM); 510 µM (NM) [100]
Glutamatergic	NR1/NR2A (NM)	Glutamate 100 µM [104]	5 nM (HAS); 79 µM (LAS) [104]
	NR1/NR2B (NM)	Glutamate 100 µM [104]	9.5 µM [104]
	GluR6R (NM)	AMPA 30–300 µM [105]	67 µM (5 min PI) [105]
	GluR6R/KA1 (NM)	AMPA 30–300 µM [105]	1.5 µM (5 min PI) [105]
	GluR6R/KA2 (NM)	AMPA 30–300 µM [105]	2.1 µM (5 min PI) [105]
	GluK3 (PM/NM)	Glutamate 10 mM [20]	46 µM (PM); 100 µM (NM) [20]
Serotoninergic	5-HT3A (PM)	5-HT 1 µM [103]	N.D. [103]
H^+^ergic	ASIC2a (PM)	pH 5 [23]	120 µM [23]
	ASIC1a (NM)	pH 6.5 [32]	7.0 nM [32]
	ASIC1a-ASIC2a (NM)	pH 6.0 [32]	10.04 nM pH 6.5 [32]
Na^+^ergic	ENaC αβγ (PM/NM)	Na^+^ 110 mM [24,106]	1.74 µM [106], 2.1 µM [24] (PM); N.D. [107], 2.1 mM [24] (NM)
Purinergic	P2X1 (NM)	ATP 0.3 µM [108]	9.34 µM, 0.82 µM (20 min PI), 1.1 µM (40 min PI) [108]
	P2X2 (PM/NM)	ATP (WC) [109], 3 µM [110], 5 µM [25], 2 µM [26], 50 µM [111]	N.D. [25,109], 9.3 µM, 6.1 µM (5 min PI) [110], 7.9 µM [26], 19.6 µM [111] (PM); N.D. (>30 µM, 5 min PI) [110] (NM)
	P2X3 (PM/NM)	ATP 0.3 µM [108]	10.9 µM (20 min PI), N.D. (<20 µM, 40 min PI) (PM); N.D. (>20 µM, 20 and 40 min PI) (NM) [108]
	P2X4 (PM/NM)	ATP 5 µM [111,112], 3 µM [113], 1 µM 25 µM [68]	N.D. (<10 µM) [112], 2.4 µM [111], 1.9 µM (0 and 15 min PI) [113], 2.4 µM, 4.9 µM (20 s PI) [68] (PM); N.D. (>100 µM) [112], N.D. (>30 µM, 0 and 15 min PI) [113] (NM)
	P2X5 (PM/NM)	ATP 300 nM [114]	42.6 µM ( 0 and 15 min PI) (PM); N.D. (0 and 15 min PI) (NM) [114]
	P2X7 (NM)	BzATP 30 µM [33,115], ATP 600 µM [34]	11.2 µM [115], 78 µM (10 s PI) [34], 4.6 µM [33]
TRP	TRPM2 (NM)	ADPR [117]	N.D. [117]
	TRPM5 (NM)	Intracellular Ca^2+^ 500 nM [118]	4.3 µM [118]
	TRPA1 (A)	–	2.3 µM [120]
Hemichannels	Hemi-gap-junction channels (NM)	0 Ca^2+^ [119]	37 µM [119]

PM, positive modulator; NM, negative modulator; A, activator; HAS, high affinity site; LAS, low affinity site; WC, without concentration; PI, preincubation; EC_50_ or IC_50_ refers to median excitatory or inhibitory concentration, respectively.

## References

[1] Maret W., Li Y. (2009). Coordination dynamics of zinc in proteins. Chem. Rev..

[2] Coddou C., Yan Z., Obsil T., Huidobro-Toro J.P., Stojilkovic S.S. (2011). Activation and regulation of purinergic P2X receptor channels. Pharmacol. Rev..

[3] Elinder F., Rhem P. (2003). Metal ion effects on ion channel gating. Q. Rev. Biophys..

[4] Coddou C., Stojilkovic S.S., Huidobro-Toro J.P. (2011). Allosteric modulation of ATP-gated P2X receptor channels. Rev. Neurosci..

[5] Andreini C., Bertini I., Cavallaro G. (2011). Minimal functional sites allow a classification of zinc sites in proteins. PLoS ONE.

[6] Auld D.S. (2001). Zinc Biochemistry, Physiology, and Homeostasis. Zinc Coordination Sphere in Biochemical Zinc Sites.

[7] Kambe T., Tsuji T., Hashimoto A., Itsumura N. (2015). The physiological, biochemical, and molecular roles of zinc transporters in zinc homeostasis and metabolism. Physiol. Rev..

[8] Laitaoja M., Valjakka J., Jänis J. (2013). Zinc coordination spheres in protein structures. Inorg. Chem..

[9] Sousa S.F., Lopes A.B., Fernandes P.A., Ramos M.J. (2009). The Zinc proteome: A tale of stability and functionality. Dalton Trans..

[10] Colvin R.A., Holmes W.R., Fontaine C.P., Maret W. (2010). Cytosolic zinc buffering and muffling: Their role in intracellular zinc homeostasis. Metallomics.

[11] Baran E.J. (1995). Química Bioinorgánica.

[12] Herrmann A.G., Blanchard D.P., Haskin L.A., Jacobs J.W., Knake D., Korotev R.L., Brannon J.C. (1976). Major, minor, and trace element compositions of peridotitic and basaltic komatiites from the precambrian crust of Southern Africa. Contrib. Mineral. Petrol..

[13] Ekpo B.O., Ibok U.J. (1998). Seasonal variation and partition of trace metals (Fe, Zn, Cu, Mn, Cr, Cd and Pb) in surface sediments: relationship with physico-chemical variables of water from the Calabar River, South Eastern Nigeria. Environ. Geochem. Health.

[14] Fisher S.Z., Kovalevsky A.Y., Domsic J.F., Mustyakimov M., McKenna R., Silverman D.N., Langan P.A. (2010). Neutron structure of human carbonic anhydrase II: Implications for proton transfer. Biochemistry.

[15] Omichinski J.G., Clore G.M., Robien M., Sakaguchi K., Appella E., Gronenborn A.M. (1992). High-resolution solution structure of the double Cys2His2 zinc finger from the human enhancer binding protein MBP-1. Biochemistry.

[16] Smith G.D., Pangborn W.A., Blessing R.H. (2003). The structure of T 6 human insulin at 1.0 Å resolution. Acta Crystallogr. Sect. D.

[17] Hallman P.S., Perrin D.D., Watt A.E. (1971). The computed distribution of copper(II) and zinc(II) ions among seventeen amino acids present in human blood plasma. Biochem. J..

[18] Alberts I.L., Nadassy K., Wodak S.J. (1998). Analysis of zinc binding sites in protein crystal structures. Protein Sci..

[19] Tamames B., Sousa S.F., Tamames J., Fernandes P.A., Ramos M.J. (2007). Analysis of zinc-ligand bond lengths in metalloproteins: Trends and patterns. Proteins Struct. Funct. Genet..

[20] Veran J., Kumar J., Pinheiro P.S., Athané A., Mayer M.L., Perrais D., Mulle C. (2012). Zinc potentiates GluK3 glutamate receptor function by stabilizing the ligand binding domain dimer interface. Neuron.

[21] Hsiao B., Mihalak K.B., Repicky S.E., Everhart D., Mederos A.H., Malhotra A., Luetje C.W. (2006). Determinants of zinc potentiation on the alpha4 subunit of neuronal nicotinic receptors. Mol. Pharmacol..

[22] Harvey R.J., Thomas P., James C.H., Wilderspin A., Smart T.G. (1999). Identification of an inhibitory Zn^2+^ binding site on the human glycine receptor α1 subunit. J. Physiol..

[23] Baron A., Schaefer L., Lingueglia E., Champigny G., Lazdunski M. (2001). Zn^2+^ and H^+^ are Coactivators of Acid-sensing Ion Channels. J. Biol. Chem..

[24] Chen J., Winarski K.L., Myerburg M.M., Pitt B.R., Sheng S. (2012). Probing the Structural Basis of Zn^2+^ Regulation of the Epithelial Na^+^ Channel. J. Biol. Chem..

[25] Clyne J.D., LaPointe L.D., Hume R.I. (2002). The role of histidine residues in modulation of the rat P2X_2_ purinoceptor by zinc and pH. J. Physiol..

[26] Nagaya N., Tittle R.K., Saar N., Dellal S.S., Hume R.I. (2005). An intersubunit zinc binding site in rat P2X_2_ receptors. J. Biol. Chem..

[27] Coddou C., Acuña-Castillo C., Bull P., Huidobro-Toro J.P. (2007). Dissecting the facilitator and inhibitor allosteric metal sites of the P2X_4_ receptor channel: Critical roles of CYS132 for zinc potentiation and ASP138 for copper inhibition. J. Biol. Chem..

[28] Li C.Y., Xiong K.M., Wu Y.X., Liu Y.W., Chen L., Stewart R.R., Peoples R.W., Yi C.L. (2013). Conserved extracellular cysteines differentially regulate the potentiation produced by Zn^2+^ in rat P2X_4_ receptors. Eur. J. Pharmacol..

[29] Fayyazuddin A., Villarroel A., Le Goff A., Lerma J., Neyton J. (2000). Four residues of the extracellular N-terminal domain of the NR2A subunit control high-affinity Zn^2+^ binding to NMDA receptors. Neuron.

[30] Zheng F., Erreger K., Low C.M., Banke T., Lee C.J., Conn P.J., Traynelis S.F. (2001). Allosteric interaction between the amino terminal domain and the ligand binding domain of NR2A. Nat. Neurosci..

[31] Wang T.-L., Hackam A., Guggino W.B., Cutting G.R. (1995). A Single histidine residue is essential for zinc inhibition of GABA. J. Neurosci..

[32] Chu X., Wemmie J.A., Wang W., Zhu X., Saugstad J.A., Price M.P., Simon R.P., Xiong Z. (2004). Subunit-dependent high-affinity zinc inhibition of acid-sensing ion channels. J. Neurosci..

[33] Liu X., Surprenant A., Mao H.-J., Roger S., Xia R., Bradley H., Jiang L.-H. (2008). Identification of key residues coordinating functional inhibition of P2X_7_ receptors by zinc and copper. Mol. Pharmacol..

[34] Acuña-Castillo C., Coddou C., Bull P., Brito J., Huidobro-Toro J.P. (2007). Differential role of extracellular histidines in copper, zinc, magnesium and proton modulation of the P2X_7_ purinergic receptor. J. Neurochem..

[35] Amin E.A., Truhlar D.G. (2008). Zn coordination chemistry: Development of benchmark suites for geometries, dipole moments, and bond dissociation energies and their use to test and validate density functionals and molecular orbital theory. J. Chem. Theory Comput..

[36] Trzaskowski B., Adamowicz L., Deymier P.A. (2008). A theoretical study of zinc(II) interactions with amino acid models and peptide fragments. J. Biol. Inorg. Chem..

[37] Kochańczyk T., Drozd A., Krężel A. (2015). Relationship between the architecture of zinc coordination and zinc binding affinity in proteins—Insights into zinc regulation. Metallomics.

[38] Burgess J., Prince R.H. (2011). Zinc: Inorganic & Coordination Chemistry. Encyclopedia of Inorganic and Bioinorganic Chemistry.

[39] Burdette S.C., Lippard S.J. (2003). Meeting of the minds: Metalloneurochemistry. Proc. Natl. Acad. Sci. USA.

[40] Hambidge K.M., Casey C.E., Krebs N.F., Mertz W. (1986). Zinc. Trace Elements in Human and Animal Nutrition.

[41] Dawson J.B., Walker B.E. (1969). Direct determination of zinc in whole blood, plasma and urine by atomic absorption spectroscopy. Clin. Chim. Acta.

[42] Frederickson C.J., Koh J., Bush A.I. (2005). The neurobiology of zinc in health and disease. Nat. Rev. Neurosci..

[43] Marger L., Schubert C.R., Bertrand D. (2014). Zinc: An underappreciated modulatory factor of brain function. Biochem. Pharmacol..

[44] Yang Y., Maret W., Vallee B.L. (2001). Differential fluorescence labeling of cysteinyl clusters uncovers high tissue levels of thionein. Proc. Natl. Acad. Sci. USA.

[45] Colvin R.A., Lai B., Holmes W.R., Lee D. (2015). Understanding metal homeostasis in primary cultured neurons. Studies using single neuron subcellular and quantitative metallomics. Metallomics.

[46] Aedo F., Delgado R., Wolff D., Vergara C. (2007). Copper and zinc as modulators of neuronal excitability in a physiologically significant concentration range. Neurochem. Int..

[47] Koeberl C., Bayer P.M., Hobarth K. (1993). Determination of Rare earth and other trace element abundances in human kidney stones and brain tissue. J. Radioanal. Nucl. Chem. Artic..

[48] Vanhoe H., Vandecasteele C., Versieck J., Dams R. (1989). Determination of iron, cobalt, copper, zinc, rubidium, molybdenum, and cesium in human serum by inductively coupled plasma mass spectrometry. Anal. Chem..

[49] Bouron A., Kiselyov K., Oberwinkler J. (2015). Permeation, regulation and control of expression of TRP channels by trace metal ions. Pflügers Arch..

[50] Kimura T., Kambe T. (2016). The Functions of metallothionein and ZIP and ZnT transporters: An overview and perspective. Int. J. Mol. Sci..

[51] Harris E.D. (2002). Cellular transporters for zinc. Nutr. Rev..

[52] Palmiter R.D., Findley S.D. (1995). Cloning and functional characterization of a mammalian zinc transporter that confers resistance to zinc. EMBO J..

[53] Palmiter R.D., Cole T.B., Findley S.D. (1996). ZnT-2, a mammalian protein that confers resistance to zinc by facilitating vesicular sequestration. EMBO J..

[54] Palmiter R.D., Cole T.B., Quaife C.J., Findley S.D. (1996). ZnT-3, a putative transporter of zinc into synaptic vesicles. Proc. Natl. Acad. Sci. USA.

[55] Huang L., Gitschier J. (1997). A novel gene involved in zinc transport is deficient in the lethal milk mouse. Nat. Genet..

[56] Kambe T., Narita H., Yamaguchi-Iwai Y., Hirose J., Amano T., Sugiura N., Sasaki R., Mori K., Iwanaga T., Nagao M. (2002). Cloning and characterization of a novel mammalian zinc transporter, zinc transporter 5, abundantly expressed in pancreatic β cells. J. Biol. Chem..

[57] Huang L., Kirschke C.P., Gitschier J. (2002). Functional characterization of a novel mammalian zinc transporter, ZnT6. J. Biol. Chem..

[58] Kirschke C.P., Huang L. (2003). ZnT7, a novel mammalian zinc transporter, accumulates zinc in the Golgi apparatus. J. Biol. Chem..

[59] Chimienti F., Devergnas S., Favier A., Seve M. (2004). Identification and cloning of a cell-specific zinc transporter, ZnT-8, localized into insulin secretory granules. Diabetes.

[60] Bosomworth H.J., Thornton J.K., Coneyworth L.J., Ford D., Valentine R.A. (2012). Efflux function, tissue-specific expression and intracellular trafficking of the Zn transporter ZnT10 indicate roles in adult Zn homeostasis. Metallomics.

[61] Grotz N., Fox T., Connolly E., Park W., Guerinot M.L., Eide D. (1998). Identification of a family of zinc transporter genes from Arabidopsis that respond to zinc deficiency. Proc. Natl. Acad. Sci. USA.

[62] Eng B.H., Guerinot M.L., Eide D., Saier M.H. (1998). Sequence analyses and phylogenetic characterization of the ZIP family of metal ion transport proteins. J. Membr. Biol..

[63] Kambe T., Yamaguchi-Iwai Y., Sasaki R., Nagao M. (2004). Overview of mammalian zinc transporters. Cell. Mol. Life Sci..

[64] Gerhart J.C., Pardee A.B. (1962). The enzymology of control by feedback inhibition. J. Biol. Chem..

[65] Monod J., Changeux J.P., Jacob F. (1963). Allosteric proteins and cellular control systems. J. Mol. Biol..

[66] Sigel E., Baur I. (1988). Allosteric modulation by benzodiazepine receptor ligands GABAA RECEPTOR channel expressed in Xenopus oocytes. J. Neurosci..

[67] Monod J., Wyman J., Changeux J.P. (1965). On the nature of allosteric transitions: A plausible model. J. Mol. Biol..

[68] Acuña-Castillo C., Morales B., Huidobro-Toro J.P. (2000). Zinc and copper modulate differentially the P2X_4_ receptor. J. Neurochem..

[69] Coddou C., Morales B., González J., Grauso M., Gordillo F., Bull P., Rassendren F., Huidobro-Toro J.P. (2003). Histidine 140 plays a key role in the inhibitory modulation of the P2X_4_ nucleotide receptor by copper but not zinc. J. Biol. Chem..

[70] Huidobro-Toro J.P., Lorca R.A., Coddou C. (2008). Trace metals in the brain: Allosteric modulators of ligand-gated receptor channels, the case of ATP-gated P2X receptors. Eur. Biophys. J..

[71] Tian C., Zhu R., Zhu L., Qiu T., Cao Z., Kang T. (2014). Potassium channels: Structures, diseases, and modulators. Chem. Biol. Drug Des..

[72] Jensen H.S., Callø K., Jespersen T., Jensen B.S., Olesen S.P. (2005). The KCNQ5 potassium channel from mouse: A broadly expressed M-current like potassium channel modulated by zinc, pH, and volume changes. Mol. Brain Res..

[73] Kim J., Park J., Kang H., Lee E.-J., Bang H., Lee J. (2005). Zinc activates TREK-2 potassium channel activity. J. Pharmacol. Exp. Ther..

[74] Spires S., Begenisich T. (1994). Modulation of potassium channel gating by external divalent cations. J. Gen. Physiol..

[75] Harrison N.L., Radke H.K., Tamkun M.M., Lovinger D.M. (1993). Modulation of gating of cloned rat and human K^+^ channels by micromolar Zn^2+^. Mol. Pharmacol..

[76] Zhang S., Kehl S.J., Fedida D. (2001). Modulation of Kv1.5 potassium channel gating by extracellular zinc. Biophys. J..

[77] Gruss M., Mathie A., Lieb W.R., Franks N.P. (2004). The two-pore-domain K^+^ channels TREK-1 and TASK-3 are differentially modulated by copper and zinc. Mol. Pharmacol..

[78] Hou S., Vigeland L.E., Zhang G., Xu R., Li M., Heinemann S.H., Hoshi T. (2010). Zn^2+^ activates large conductance Ca^2+^-activated K^+^ channel via an intracellular domain. J. Biol. Chem..

[79] Gilly W.F., Armstrong C.M. (1982). Slowing of sodium channel opening kinetics in squid axon by extracellular zinc. J. Gen. Physiol..

[80] Kuo C.-C., Chen W.-Y., Yang Y.-C. (2004). Block of tetrodotoxin-resistant Na^+^ channel pore by multivalent cations: Gating modification and Na^+^ flow dependence. J. Gen. Physiol..

[81] Simms B.A., Zamponi G.W. (2014). Neuronal voltage-gated calcium channels: Structure, function, and dysfunction. Neuron.

[82] Traboulsie A., Chemin J., Chevalier M., Quignard J.-F., Nargeot J., Lory P. (2007). Subunit-specific modulation of T-type calcium channels by zinc. J. Physiol..

[83] Sun H.-S., Hui K., Lee D.W.K., Feng Z.-P. (2007). Zn^2+^ sensitivity of high- and low-voltage activated calcium channels. Biophys. J..

[84] Park S., Min S., Kang H., Lee J. (2015). Differential zinc permeation and blockade of L-type Ca^2+^ channel isoforms Ca_v_1.2 and Ca_v_1.3. Biochim. Biophys. Acta.

[85] Yuan H., Gao C., Chen Y., Jia M., Geng J., Zhang H., Zhan Y., Boland L.M., An H. (2013). Divalent cations modulate TMEM_1_6A calcium-activated chloride channels by a common mechanism. J. Membr. Biol..

[86] De Coursey T.E. (2013). Voltage-gated proton channels: Molecular biology, physiology, and pathophysiology of the H(V) family. Physiol. Rev..

[87] Mahaut-Smith M.P. (1989). The effect of zinc on calcium and hydrogen ion currents in intact snail neurones. J. Exp. Biol..

[88] Ramsey I.S., Moran M.M., Chong J.A., Clapham D.E. (2006). A voltage-gated proton-selective channel lacking the pore domain. Nature.

[89] Musset B., Smith S.M.E., Rajan S., Cherny V.V., Sujai S., Morgan D., De Coursey T.E. (2010). Zinc inhibition of monomeric and dimeric proton channels suggests cooperative gating. J. Physiol..

[90] Verkman A.S., Galietta L.J.V. (2009). Chloride channels as drug targets. Nat. Rev. Drug Discov..

[91] Stölting G., Fischer M., Fahlke C. (2014). CLC channel function and dysfunction in health and disease. Front. Physiol..

[92] Chen T.Y. (1998). Extracellular zinc ion inhibits ClC-0 chloride channels by facilitating slow gating. J. Gen. Physiol..

[93] Davies P.A., Wang W., Hales T.G., Kirkness E.F. (2003). A novel class of ligand-gated ion channel is activated by Zn^2+^. J. Biol. Chem..

[94] Bloc A., Cens T., Cruz H., Dunant Y. (2000). Zinc-induced changes in ionic currents of clonal rat pancreatic -cells: Activation of ATP-sensitive K^+^ channels. J. Physiol..

[95] Prost A., Bloc A., Hussy N., Derand R., Vivaudou M. (2004). Zinc is both an intracellular and extracellular regulator of KATP channel function. J. Physiol..

[96] Ruiz A., Walker M.C., Fabian-Fine R., Kullmann D.M. (2004). Endogenous zinc inhibits GABAA receptors in a hippocampal pathway. J. Neurophysiol..

[97] Feigenspan A., Weiler R. (2004). Electrophysiological properties of mouse horizontal cell GABAA receptors. J. Neurophysiol..

[98] Calvo D.J., Vazquez A.E., Miledi R. (1994). Cationic modulation of rho 1-type γ-aminobutyrate receptors expressed in Xenopus oocytes. Proc. Natl. Acad. Sci. USA.

[99] Chang Y., Amin J., Weiss D.S. (1995). Zinc is a mixed antagonist of homomeric rho 1 γ-aminobutyric acid-activated channels. Mol. Pharmacol..

[100] Hsiao B., Dweck D., Luetje C.W. (2001). Subunit-dependent modulation of neuronal nicotinic receptors by zinc. J. Neurosci..

[101] Palma E., Maggi L., Miledi R., Eusebi F. (1998). Effects of Zn^2+^ on wild and mutant neuronal α7 nicotinic receptors. Proc. Natl. Acad. Sci. USA.

[102] Ohno Y., Shimizu S., Tokudome K. (2013). Pathophysiological roles of serotonergic system in regulating extrapyramidal motor functions. Biol. Pharm. Bull..

[103] Hubbard P.C., Lummis S.C.R. (2000). Zn^2+^ enhancement of the recombinant 5-HT3 receptor is modulated by divalent cations. Eur. J. Pharmacol..

[104] Chen N., Moshaver A., Raymond L.A. (1997). Differential sensitivity of recombinant *N*-methyl-d-aspartate receptor subtypes to zinc inhibition. Mol. Pharmacol..

[105] Mott D.D., Benveniste M., Dingledine R.J. (2008). pH-Dependent inhibition of kainate receptors by zinc. J. Neurosci..

[106] Sheng S., Perry C.J., Kleyman T.R. (2004). Extracellular Zn^2+^ activates epithelial Na^+^ channels by eliminating Na^+^ self-inhibition. J. Biol. Chem..

[107] Kellenberger S., Gautschi I., Pfister Y., Schild L. (2005). Intracellular thiol-mediated modulation of epithelial sodium channel activity. J. Biol. Chem..

[108] Wildman S.S., King B.F., Burnstock G. (1999). Modulatory activity of extracellular H^+^ and Zn^2+^ on ATP-responses at rP2X_1_ and rP2X_3_ receptors. Br. J. Pharmacol..

[109] Nakazawa K., Ohno Y. (1997). Effects of neuroamines and divalent cations on cloned and mutated ATP-gated channels. Eur. J. Pharmacol..

[110] Wildman S.S., King B.F., Burnstock G. (1998). Zn^2+^ modulation of ATP-responses at recombinant P2X_2_ receptors and its dependence on extracellular pH. Br. J. Pharmacol..

[111] Xiong K., Peoples R.W., Montgomery J.P., Chiang Y., Stewart R.R., Weight F.F., Li C. (1999). Differential modulation by copper and zinc of P2X_2_ and P2X_4_ receptor function. J. Neurophysiol..

[112] Garcia-Guzman M., Soto F., Gomez-Hernandez J.M., Lund P.E., Stühmer W. (1997). Characterization of recombinant human P2X_4_ receptor reveals pharmacological differences to the rat homologue. Mol. Pharmacol..

[113] Wildman S.S., King B.F., Burnstock G. (1999). Modulation of ATP-responses at recombinant rP2X_4_ receptors by extracellular pH and zinc. Br. J. Pharmacol..

[114] Wildman S.S., Brown S.G., Rahman M., Noel C.A., Churchill L., Burnstock G., Unwin R.J., King B.F. (2002). Sensitization by extracellular Ca^2+^ of rat P2X_5_ receptor and its pharmacological properties compared with rat P2X_1_. Mol. Pharmacol..

[115] Virginio C., Church D., North R.A., Surprenant A. (1997). Effects of divalent cations, protons and calmidazolium at the rat P2X_7_ receptor. Neuropharmacology.

[116] Flockerzi V., Nilius B., Nilius B., Flockerzi V. (2014). Mammalian transient receptor potential (TRP) cation channels. Handbook of Experimental Pharmacology.

[117] Yang W., Manna P.T., Zou J., Luo J., Beech D.J., Sivaprasadarao A., Jiang L.H. (2011). Zinc inactivates melastatin transient receptor potential 2 channels via the outer pore. J. Biol. Chem..

[118] Uchida K., Tominaga M. (2013). Extracellular zinc ion regulates transient receptor potential melastatin 5 (TRPM5) channel activation through its interaction with a pore loop domain. J. Biol. Chem..

[119] Sun Z., Zhang D., McMahon D.G. (2009). Zinc modulation of hemi-gap-junction channel currents in retinal horizontal cells. J. Neurophysiol..

[120] Hu H., Bandell M., Petrus M.J., Zhu M.X., Patapoutian A. (2009). Zinc activates damage-sensing TRPA1 ion channels. Nat. Chem. Biol..

[121] Yu P., Wang Q., Zhang L.H., Lee H.C., Zhang L., Yue J. (2012). A cell permeable NPE caged ADP-Ribose for Studying TRPM2. PLoS ONE.

[122] Inoue K., Branigan D., Xiong Z.G. (2010). Zinc-induced neurotoxicity mediated by transient receptor potential melastatin 7 channels. J. Biol. Chem..

[123] Shuart N.G., Haitin Y., Camp S.S., Black K.D., Zagotta W.N. (2011). Molecular mechanism for 3:1 subunit stoichiometry of rod cyclic nucleotide-gated ion channels. Nat. Commun..

[124] Bixby K.A., Nanao M.H., Shen N.V., Kreusch A., Bellamy H., Pfaffinger P.J., Choe S. (1999). Zn^2+^-binding and molecular determinants of tetramerization in voltage-gated K^+^ channels. Nat. Struct. Biol..

[125] Rotter I., Kosik-Bogacka D., Dołęgowska B., Safranow K., Lubkowska A., Laszczyńska M. (2015). Relationship between the concentrations of heavy metals and bioelements in aging men with metabolic syndrome. Int. J. Environ. Res. Public Health.

[126] Watt N.T., Whitehouse I.J., Hooper N.M. (2011). The role of zinc in Alzheimer’s disease. Int. J. Alzheimers Dis..

[127] Morris D.R., Levenson C.W. (2012). Ion channels and zinc: Mechanisms of neurotoxicity and neurodegeneration. J. Toxicol..

[128] Li S.-O., Wang J.-L., Bjørklund G., Zhao W.-N., Yin C.-H. (2014). Serum copper and zinc levels in individuals with autism spectrum disorders. Neuroreport.

[129] Macedoni-Lukšič M., Gosar D., Bjørklund G., Oražem J., Kodrič J., Lešnik-Musek P., Zupančič M., France-Štiglic A., Sešek-Briški A., Neubauer D. (2015). Levels of metals in the blood and specific porphyrins in the urine in children with autism spectrum disorders. Biol. Trace Elem. Res..

[130] Wyatt L.R., Godar S.C., Khoja S., Jakowec M.W., Alkana R.L., Bortolato M., Davies D.L. (2013). Sociocommunicative and sensorimotor impairments in male P2X_4_-deficient mice. Neuropsychopharmacology.

[131] Nickols H.H., Conn J.P. (2014). Development of allosteric modulators of GPCRs for treatment of CNS disorders. Neurobiol. Dis..

[132] Lorca A., Rozas C., Loyola S., Moreira-ramos S., Zeise M.L., Kirkwood A., Huidobro-Toro J.P., Morales B. (2011). Zinc enhances long-term potentiation through P2X receptor modulation in the hippocampal CA1 region. Eur. J. Neurosci..

[133] Xie Y., Wang Y., Zhang T., Ren G., Yang Z. (2012). Effects of nanoparticle zinc oxide on spatial cognition and synaptic plasticity in mice with depressive-like behaviors. J. Biomed. Sci..

